# Mitochondrial DNA density homeostasis accounts for a threshold effect in a cybrid model of a human mitochondrial disease

**DOI:** 10.1042/BCJ20170651

**Published:** 2017-11-24

**Authors:** Juvid Aryaman, Iain G. Johnston, Nick S. Jones

**Affiliations:** 1 Department of Mathematics, Imperial College London, London, U.K.; 2 School of Biosciences, University of Birmingham, Birmingham, U.K.

**Keywords:** MELAS, mitochondria, mitochondrial dysfunction, mtDNA, threshold effect

## Abstract

Mitochondrial dysfunction is involved in a wide array of devastating diseases, but the heterogeneity and complexity of the symptoms of these diseases challenges theoretical understanding of their causation. With the explosion of omics data, we have the unprecedented opportunity to gain deep understanding of the biochemical mechanisms of mitochondrial dysfunction. This goal raises the outstanding need to make these complex datasets interpretable. Quantitative modelling allows us to translate such datasets into intuition and suggest rational biomedical treatments. Taking an interdisciplinary approach, we use a recently published large-scale dataset and develop a descriptive and predictive mathematical model of progressive increase in mutant load of the MELAS 3243A>G mtDNA mutation. The experimentally observed behaviour is surprisingly rich, but we find that our simple, biophysically motivated model intuitively accounts for this heterogeneity and yields a wealth of biological predictions. Our findings suggest that cells attempt to maintain wild-type mtDNA density through cell volume reduction, and thus power demand reduction, until a minimum cell volume is reached. Thereafter, cells toggle from demand reduction to supply increase, up-regulating energy production pathways. Our analysis provides further evidence for the physiological significance of mtDNA density and emphasizes the need for performing single-cell volume measurements jointly with mtDNA quantification. We propose novel experiments to verify the hypotheses made here to further develop our understanding of the threshold effect and connect with rational choices for mtDNA disease therapies.

## Introduction

Mitochondria are organelles which are present across much of eukaryotic life and are known for their role in the production of ATP, the major energy currency of the cell. In humans, mitochondrial dysfunction is associated with a host of diseases because of the role of mitochondria in metabolism, biosynthesis [1] and energy supply, as well as their importance in cell death signalling [2], implicating them in diseases ranging from neurodegeneration [3] to cancer [4]. Fundamental understanding of these organelles and their dysfunction is, therefore, of far-reaching biomedical importance.

Mitochondria generate ATP by pumping electrons across their inner membrane to generate an electrochemical gradient, which is used by ATP synthase to convert ADP into ATP. The process of electron pumping is known as the electron transport chain (ETC), and this pathway of ATP generation is called oxidative phosphorylation (OXPHOS). Mitochondria also possess their own circular DNA (mtDNA), which are held in multiple copies per cell. In humans, these genomes encode 13 proteins (which encode subunits of complexes I, III and IV of the ETC and ATP synthase), 22 tRNAs and 2 rRNAs. An important class of diseases which affect mitochondria are those which are caused by a mutation in mtDNA. The most common, and most studied, of these is MELAS (mitochondrial encephalomyopathy, lactic acidosis, and stroke-like episodes) syndrome, which is often associated with a mitochondrial tRNA mutation at position 3243A>G of the mitochondrial genome [5,6]. Its incidence rate shows large regional variability, with a prevalence of 1 : 6000 in Finland [7] to 1 : 424 in Australia [8]. tRNAs affected by the mutation cause amino acid misincorporations during translation and generate defective mitochondrial proteins, inducing defective respiration when mutant load (or heteroplasmy, which is the ratio of the mutant mtDNA copy number to the total mtDNA copy number per cell) is high [9].

A common feature in many diseases associated with mutations of mitochondrial DNA, including MELAS, is the non-linear physiological response of cells and tissues to increasing levels of mtDNA heteroplasmy. In particular, cells appear to be able to withstand high levels of heteroplasmy without showing any significant metabolic or physiological defect. For instance, fibroblasts possessing the MELAS mutation were shown to have unaffected respiratory enzyme activity until mutant load exceeded ∼60% [10]. Also, Chomyn et al. [11] showed that oxygen consumption of cells does not significantly reduce until MELAS heteroplasmy exceeds ∼90%. This observation has been named the threshold effect (reviewed in ref. [12]).

It has been argued that the threshold effect occurs because mitochondria possess spare capacity at the translational, enzymatic and biochemical levels, which are each able to absorb some degree of stress and thus delay the phenotypic response of increasing heteroplasmy, until a particular threshold heteroplasmy is exceeded, which is typically large [12]. Within this picture, each physiological feature (such as enzymatic activity or oxygen consumption) may be expected to display step-like behaviour with respect to increasing heteroplasmy. Asynchrony of thresholds between different features, such as 60% for enzyme activity [10] and 90% for oxygen consumption [11], may be explained by spare capacity at intermediate levels. For example, a biochemical threshold effect could account for asynchrony in oxygen consumption and enzyme activity thresholds, where metabolic fluxes are altered to compensate for fewer functional enzymes and hence prolong an oxygen consumption deficit to higher levels of enzymatic dysfunction [12].

A recent study published by Picard et al. [13] established 143B TK^−^ cybrid osteosarcoma cell lines containing the MELAS 3243A>G mutation across the full-dose response of mutant load. These cybrid cell lines were generated via the transfer of mtDNAs from a lymphoblastoid cell line, derived from a heteroplasmic 3243A>G patient, into 143B TK^−^ mtDNA-deficient cells. Although cybrid models such as this have had many non-physiological perturbations made to them, the advantage in using this system is that all cells possess the same nuclear background, and can be generated with heteroplasmies across the full spectrum of mutant load. The authors measured a diverse array of features including RNA expression, protein expression, cell volume, growth rates, mitochondrial morphology and mtDNA content. The sheer diversity of data collected, across multiple levels of heteroplasmy, makes this an important dataset in understanding the threshold effect and mitochondrial dysfunction (despite the fact that the experiment consists of a single biological replicate, albeit with several technical replicates). Under the interpretation of the threshold effect presented above, one might expect a monotonic response to heteroplasmy, as spare capacity is depleted and the cell seeks alternative means of energy provision. However, Picard et al. [13] observed complex multiphasic responses across numerous physiological readouts as heteroplasmy was increased.

The authors of the study identified four distinct transcriptional phases in the gene expression profiles of MELAS 3243A>G cells: 0%, 20–30%, 50–90% and 100% mutant load. They argue that continuous changes in heteroplasmy result in discrete changes in phenotype, because there exist a limited number of states that the nucleus can acquire in response to progressive changes in retrograde signalling [13]. In this work, by considering a distilled subset of the data from ref. [13], and using simple, physically motivated arguments, we attempt to provide a simplified account of this dataset to gain better understanding of the consequences of this mutation and the threshold effect.

Our mathematical model suggests that cells attempt to maintain homeostasis in wild-type mtDNA density at low heteroplasmies, through reduction in cell volume and therefore cellular power demand. We propose the existence of a single critical heteroplasmy, where cells are no longer able to maintain wild-type mtDNA density homeostasis, and toggle from power demand reduction to supply increase. In this regime, energy supply pathways are up-regulated. Our model also identifies an additional bioenergetic transition, in excess of 90% mutant load, as cells become fully homoplasmic. We explore the possibility of reduced transcriptional activity in mutant mtDNAs/mitochondria, limited tRNA diffusivity and a connection between cellular proliferation rate and cell volume, finding all of the above to have explanatory power. We propose new experiments to verify the novel hypotheses made here to drive forward understanding of the threshold effect.

## Materials and methods

### Data normalization

**Figure 1.**
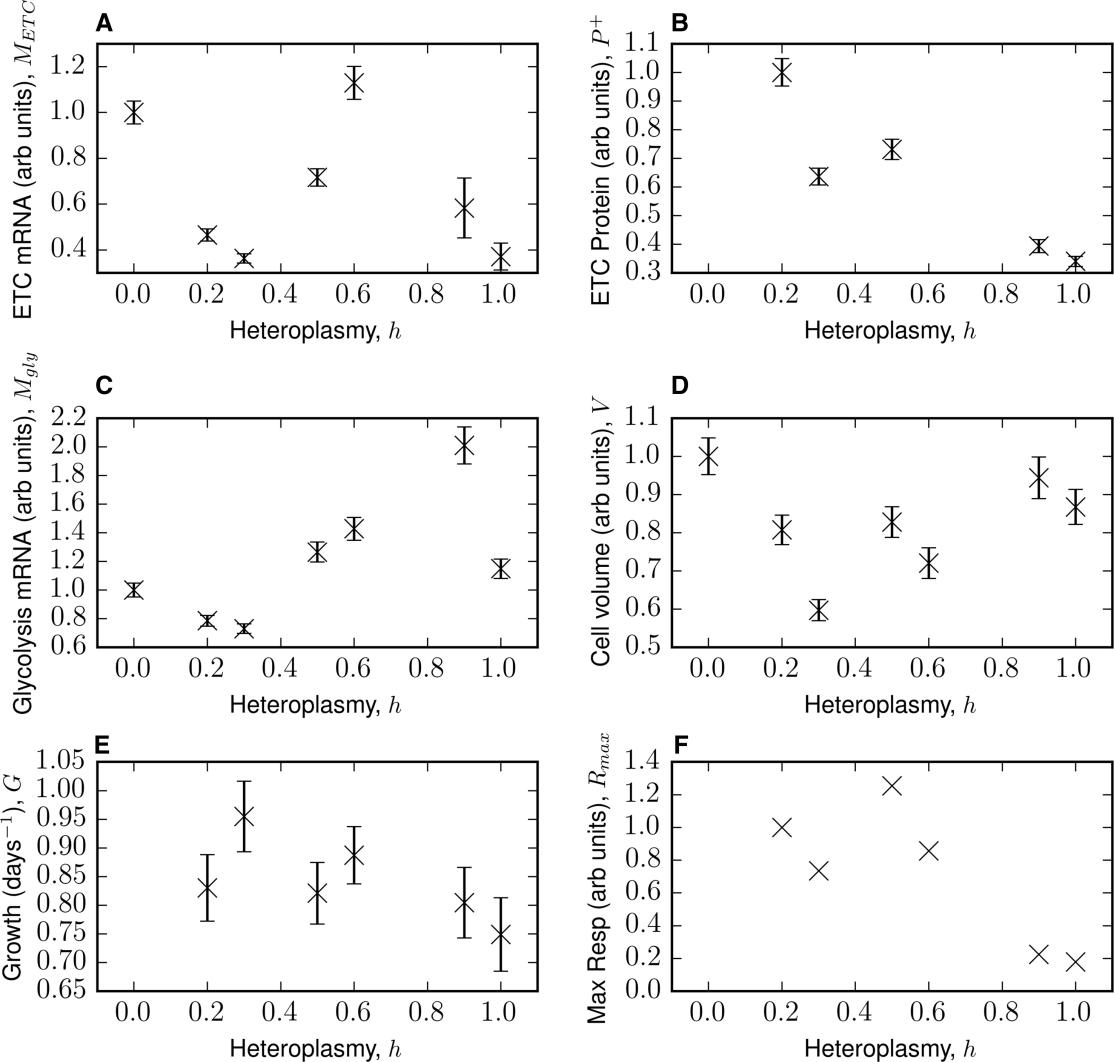
Multiphasic physiological response to increasing heteroplasmy — core data considered from Picard et al. renormalized to be in per-cell dimensions. Selected measurements of 143B TK^−^ osteosarcoma cells heteroplasmic in MELAS 3243A>G, from ref. [13]. (**A**) mRNA levels for 11 mitochondrially encoded ETC subunits (COX3, ND2, ND5, CYTB, ND3, ND6, COX1, ND4, COX2, ND1 and ND4L). (**B**) Protein levels for complexes I, III and IV. (**C**) Glycolysis mRNA levels, for genes (PKM2, ENO1, PGAM4, PGK1, GAPDH, ALDOA, PFKP, GPI, HK2 and SLC2A1). All errors in **A–C** are the standard error of the transformed renormalized mean [see eqn (2)]. (**D**) Mean cell volume of an asynchronous population of growing cells ± SEM. (**E**) Growth rate, determined by linear regression. Error is the standard error in the slope from linear regression. (**F**) Maximum respiratory capacity. Details of the data normalization and regression may be found in Materials and Methods. See ref. [13] for experimental protocols.

We considered a core subset of six physiological features from the dataset of ref. [13] for mathematical modelling: mitochondrially encoded ETC mRNA pool size, ETC protein levels, glycolysis mRNA pool size, cell volume, cell proliferation rate (or cell growth rate as termed in ref. [13]) and maximal respiratory capacity. We performed many normalizations to the data, the result of which is shown in Figure 1.

OXPHOS, as well as glycolysis, have multiple different mRNAs participating in their respective pathways. To have some measure of the overall expression level of a pathway $\left(\bar{E}\right)$, we used the mRNA concentration (in RPMK, reads per kilobase of transcript per million mapped reads), for each gene corresponding to enzymes of the pathway (*e_i__,__k_*(*h*), for gene *i* and technical replicate *k* at heteroplasmy *h*) and took a normalized sum as follows:

1 $$\bar{E}=\frac{1}{N}\frac{1}{n_{r}}\overset{n_{r}}{\underset{k=1}{\sum }}\sum_{i=1}^{N}\frac{e_{i,k}\left(h\right)}{1/n_{r}\left[\sum_{l=1}^{n_{r}}e_{i,l}\left(h=0\right)\right]}\;,$$

where *n*_r_ = number of technical replicates and *N* = number of genes in the pathway of interest. This quantity normalizes the expression level of each gene to mean *h* = 0 levels, to avoid effects from consistently highly expressed genes. The factor of 1/*N* results in $\bar{E}$ having the value of 1 at *h* = 0, so may be interpreted as a fold-change in expression relative to *h* = 0.

The standard error of $\bar{E}$ over technical replicates *k* may be written as follows:

2 $$s_{\bar{E}}=\frac{1}{N}\frac{1}{\sqrt{n_{r}}}{\left(V_{k}\left[\overset{N}{\underset{i=1}{\sum }}\frac{e_{i,k}\left(h\right)}{1/n_{r}\left[\sum_{l=1}^{n_{r}}e_{i,l}\left(h=0\right)\right]}\right]\right)}^{1/2}\;,$$

where *V*_*k*_(*x*_*k*_) is the sample variance over *x_k_*. Eqns (1) and (2) are applied to glycolysis and ETC mRNAs in our main model, which yield dimensionless, normalized, measures of transcript levels for each biological pathway.

ATP synthase was excluded from both ETC mRNA and protein data, as it is expected to be regulated differently from other ETC proteins. This difference arises because mitochondrial membrane potential is required for cell growth [14], and glycolytic ATP may be used, even in cells without mtDNA, by ATP synthase to maintain membrane potential [15]. Thus, protein levels of ATP synthase may be expected to be regulated quite differently to those of the ETC, and not generally indicative of respiratory activity.

For ETC protein, we simply used the sample mean of complexes I, III and IV, since the data given by Picard et al. [13] are already normalized.

### Data transformation to per-cell dimensions

The data we considered of Picard et al. [13] consist of RNA-seq and Western blot measurements for mRNA and protein levels, respectively. We wished to model the bioenergetic strategy of an average cell, so it is important that the data used to parameterize the model are of per-cell dimensions. We show in Supplementary Text S1 that it is appropriate to multiply protein and transcript data by cell volume to gain per-cell dimensions.

### Error propagation

Our work focuses on describing the mean behaviour of various cellular processes with respect to heteroplasmy, so uncertainty in these means must be quantified. For *M*_gly_ and *M*_ETC_, we used error propagation on the normalized transcript levels $\bar{E}$ [see eqn (1)] and *V*, to derive the volume-adjusted transcript uncertainties for the data shown in Figure 1

3 $$\sqrt{{\bar{E}}^{2}s_{V}^{2}+V^{2}s_{\bar{E}}^{2}}$$

where $s_{\bar{E}}$ is defined in eqn (2) and *s_V_* is the SEM for cell volume (raw data provided by Martin Picard). For the case of ETC protein data, since the corresponding experiments in Picard et al. [13] had only a single technical replicate, we derived an uncertainty by simply multiplying the normalized protein value (see ‘Data Normalization’) by *s_V_*.

### Growth rate determination

The speed with which cells proliferate is dependent on heteroplasmy, as can be seen in Supplementary Figure S1. However, by day 6 of growth, cell growth appeared to change its behaviour, with evidence of saturation; we, therefore, truncated the raw data to day 5 and calculated the exponential growth rate by linear regression in log-lin space.

### Model inference

We used Bayesian inference to determine the parametric uncertainty in the mathematical model presented below, given the data in ref. [13], for the six features considered here. We assumed Gaussian noise for the error in the normalized versions of each feature. For full details of the priors used for each feature, see Supplementary Text S2.

## Results

### Per-cell interpretation of omics data highlights multiphasic dynamics in response to heteroplasmic load

A simple interpretation of the threshold effect predicts the existence of spare capacity in the transcription, translation, enzyme complex and biochemical levels of the cell, in response to increasing heteroplasmy [12]. Under this interpretation of the threshold effect, we might expect all of these functions to have no more than one turning point with increasing heteroplasmy.

However, the data in Figure 1, and indeed the dataset of Picard et al. [13] overall, show a much more complex response. For instance, ETC transcripts clearly show two turning points, suggesting some kind of transient compensatory response. Across the features, these data also appear to be asynchronous in their turning points. For instance, ETC transcripts peak at *h* = 0.6, but glycolysis transcripts peak at *h* = 0.9. This highlights the need for an extension in our understanding of the threshold effect, as well as the challenge in trying to parsimoniously model such a complex dataset.

We note that the measurement uncertainty, reported in [13], for our selected features of interest is small relative to the variation with respect to heteroplasmy (see Figure 1), justifying a non-linear fit to the data. It should be noted, however, that this uncertainty only reflects the technical variability in measurement and does not include potential biological variability of these features. We use a Bayesian approach to appropriately account for this uncertainty (see Supplementary Text S2).

### Integrated omics data motivate a model of the causal relationships between bioenergetic variables

**Figure 2.**
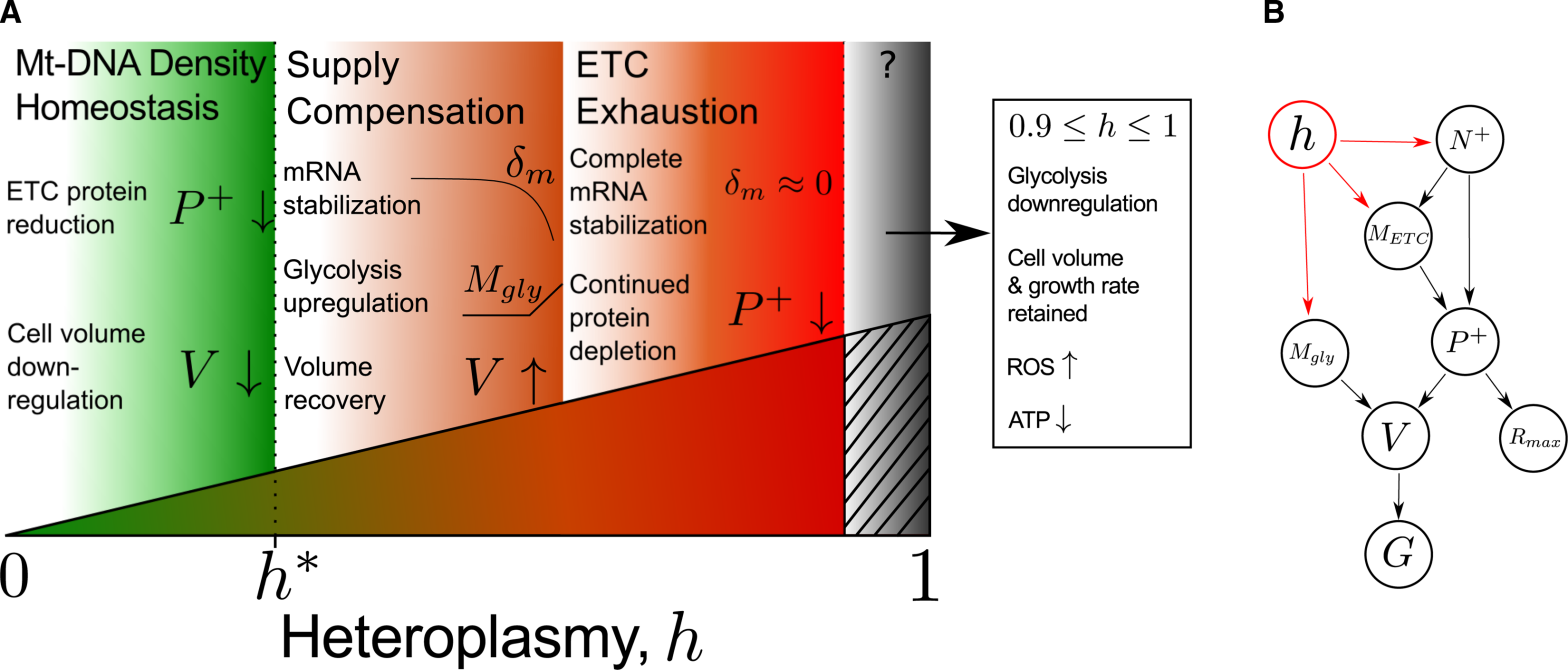
Qualitative description of continuous increase in MELAS mutant load. (**A**) At low levels of heteroplasmy (*h*), cells attempt to maintain homeostasis in wild-type mtDNA density by reducing their volume (*V*). This reduces power demands, allowing power supply/demand balance to be maintained despite rising heteroplasmy. A further increase in heteroplasmy triggers a power demand/supply toggle at a critical heteroplasmy h*, where energy provision pathways are up-regulated. This includes up-regulation of both oxidative phosphorylation, by reduction in mRNA degradation (*δ*_m_), and glycolysis transcripts (*M*_gly_). Cell volume consequently recovers. A further increase in heteroplasmy exhausts ETC stabilization, as *δ*_m_ → 0, and ETC protein (*P*^+^) continues to deplete. In the transition to homoplasmy, glycolysis and ATP levels reduce, yet cell volume and growth rate are retained. In this regime, the mode of energy production is unclear. (**B**) Flow of causality in our mathematical model. *N*^+^, wild-type mtDNA copy number; *M*_ETC_, ETC mRNA; *R*_max_, maximum respiratory capacity; *G*, cellular growth rate. Heteroplasmy shown in red connecting to variables with explicit dependence.

We present a qualitative description of our model in Figure 2, which we will develop into a full quantitative description below. One of our central claims is the existence of a single transition in cellular behaviour, in response to increasing heteroplasmy of the 3243A>G mutation, over the 0–90% heteroplasmy range. We propose that, at low heteroplasmy values, *cells attempt to maintain homeostasis in wild-type mtDNA density by reducing their volume*. This reduces biosynthetic and translational power demands, by the simplifying assumption that power demand scales directly with cell volume.

Our model suggests that at a critical heteroplasmy, h*, cells undergo a demand/supply toggle where power supply is up-regulated. ETC transcripts are stabilized through reduced degradation, and glycolysis is increased. This bioenergetic compensatory behaviour at intermediate heteroplasmies allows cell volume to recover.

As heteroplasmy continues to increase, we claim that degradation of ETC transcripts becomes negligible. Thus, further increases in heteroplasmy result in reduction in ETC protein content and ETC exhaustion ensues.

These behaviours are captured in the mathematical framework of our model. However, as cells transition from 90 to 100% mutant mtDNA, another transition in cellular behaviour appears to occur, according to the data of ref. [13]. Cells down-regulate glycolysis, yet retain cell volume and growth rate. The mode of energy production in this case is unclear and opens new questions as to the most relevant energy supplies and demands in homoplasmic cells (see ‘Key Claims and Predictions of Biophysical Model of Heteroplasmy’).

### Interactions between bioenergetic variables can be cast as a bottom-up quantitative model

We now present a quantitative description of our model, see Table 1, whose mechanistic interpretations will be more fully explored in the section ‘Key Claims and Predictions of Biophysical Model of Heteroplasmy’. Our model attempts to unify the experimentally measured features of ref. [13] within a simple, physically plausible, bottom-up cell biological representation. We stress that our choice of model structure was not developed independently of the data in ref. [13]; hence, at the level of choice of model structure, we have limited control of over-fit. However, the uncertainty in its parameters, given the data and a set of priors (see Supplementary Text S2), was computed using Bayesian inference. So, while our parameterization of the model has statistical control for uncertainty, we have not employed a statistical model selection framework. We believe this to be appropriate and practically unavoidable, as our objective is to yield a new reduced account for these heterogeneous data, present novel hypotheses and propose new experiments.

**Table 1:** Mathematical model of MELAS 3243 A>G mutation with progressive mutant load See Supplementary Table S1 for parameter descriptions. Heteroplasmy = *h*, mtDNA copy number = *N*.

Description	Equation	Equation numbers
Wild-type mtDNA, *N*^+^	$N^{+}=N\left(1-h\right)$	(4)
ETC mRNA, *M*_ETC_	$M_{\mathrm{ETC}}=\frac{\beta }{\delta_{m}+1}N^{+}$	(5)
	$\delta_{m}\left(h\right)=\frac{k_{\mathrm{mRNA}}}{1+\exp\left[k_{m}\left(h-h_{0}\right)\right]}$	(6)
ETC protein, *P*^+^	$P^{+}=\frac{N^{+}M_{\mathrm{ETC}}}{\delta_{p}}$	(7)
Glycolysis mRNA, *M*_gly_	$M_{\mathrm{gly}}=\left\{\begin{array}{c} c_{1},\;\;\;h\leq h^{*}\;\\ m_{2}h+c_{2},\;\;\;h>h^{*}\; \end{array}\right.$	(8)
Cell volume, *V*	$\underset{\mathrm{supply}}{\underbrace{k_{o}P^{+}+k_{g}M_{\mathrm{gly}}}}=\underset{\mathrm{demand}}{\underbrace{V}}$	(9)
Growth rate, *G*	$G=\frac{k_{\mathrm{gr}}}{V}$	(10)
Maximum respiratory capacity, *R*_max_	$R_{\max}=k_{p}P^{+}$	(11)

#### Wild-type mtDNA scaling

A theme apparent in the data of Picard et al. is an overall downward trend of ETC mRNA and ETC protein with increasing heteroplasmy (*h*). We, therefore, use the hypothesis that these quantities scale with the amount of wild-type mtDNA (*N*^+^). Total mtDNA copy number showed only a weak dependence on heteroplasmy, with only one heteroplasmy value displaying statistically significant deviation from *h* = 0 copy number (Figure 1I of [13]). We, therefore, assume that *N* = mtDNA copy number = const (set to 1 after normalization, without loss of generality) which then defines *N*^+^, see eqn (4). The successful performance of this simple model for *N*^+^ is shown in Supplementary Figure S2. Note that this model has no free parameters, so was not associated with our inferred posteriors.

#### ETC mRNA

Transcript copy number is determined by the balance of transcription (*β*) and degradation (*δ*_m_) rates. Given our assumption of *N*^+^ scaling, it can be shown (see Supplementary Text S3) that eqn (5) may be used to model the ETC mRNA pool size (*M*_ETC_). We further assume a constant mean transcription rate *β* for parsimony and allow the degradation rate *δ*_m_ to vary with heteroplasmy in response to cellular signals. We require the degradation rate to be high for low heteroplasmies, and low at high heteroplasmies, to describe the ability of cells to up-regulate their transcript copy number with rising heteroplasmy. A biologically motivated choice of function which achieves this is a sigmoid, see eqn (6), where *k*_mRNA_, *k*_m_ and *h*_0_ are constants.

#### ETC protein

It is intuitive to assume that mean protein levels scale with transcript levels, although this relationship may be noisy [16]. Following a similar assumption for ETC mRNA, we also assume that ETC protein (*P*^+^) scales with wild-type mtDNA levels. Using analogous arguments to *M*_ETC_ (see Supplementary Text S3), we show that a reasonable model for ETC protein is eqn (7), where *δ*_p_ = const, denoting the baseline degradation of mitochondrial protein.

#### Glycolysis mRNA

We assume that the glycolysis mRNA pool size (*M*_gly_) is invariant to heteroplasmy, until a critical heteroplasmy h*, where glycolysis is gradually up-regulated as a result of cellular control. It is, therefore, parsimonious to assume that glycolysis regulation obeys a spline of a constant and linear model which toggles at h*, see eqn (8), where *c*_1_ and *m*_2_ are free parameters, and *c*_2_ = *c*_1_ − *m*_2_h*, by continuity.

#### Cell volume

We propose that the power demands of the cell may be well approximated as scaling with cell volume (see Supplementary Text S3 for further discussion). As glycolysis and OXPHOS provide power supply to a first approximation, we assume that mean cell volume in an asynchronous population of cells (*V*) is effectively determined by a scaled sum of glycolysis and OXPHOS contributions, such that the cell obeys a power supply = power demand relationship, see eqn (9), where *k*_o_, *k*_g_ = const. We propose that changes in cellular volume are a long-term adaptation to heteroplasmy and therefore power availability.

From Figure 1, it is clear that this assumption fails at *h* = 1, where glycolysis levels and cell volume are comparable to *h* = 0 levels, and yet ETC proteins are only 30% of wild-type levels. As ATP levels are below wild-type levels in these cells (see Supplementary Figure S6E in [13]), the mode of energy production is not clear and further metabolomic data may be required. We, therefore, exclude all *h* = 1 data and limit the domain of our model to 0 ≤ *h* ≤ 0.9.

#### Growth rate

We observe that the cellular proliferation rate (which we call growth rate, *G*, for consistency with ref. [13]) varies with heteroplasmy (see Figure 1). We hypothesize that there exists a relationship between mean cell volume and growth rate. It can be shown that, assuming individual cells increase their volume linearly through the cell cycle, growth rate varies inversely with mean cell volume (shown in Supplementary Text S3). This is shown in eqn (10), where *k*_gr_ is a constant.

We show in Supplementary Text S3 that, under an exactly exponential model of cell growth, *G* is independent of *V*. However, given that there is presumably a wide class of cytoplasmic growth-vs.-time profiles which cells may obey, we use a linear model as a parsimonious example of how cell growth may be connected to cytoplasmic volume.

#### Maximum respiratory capacity

It has long been recognized that cells carrying the MELAS mutation experience a respiratory defect when heteroplasmic load exceeds ∼90% [11,17], and that this is due to a defect in protein synthesis [9,11]. We, therefore, assume that maximum respiratory capacity (*R*_max_) is always determined by protein content. This yields a simple linear expression, see eqn (11), where *k*_p_ = const.

#### Model summary

In summary, our model of mean cellular behaviour with respect to heteroplasmy describes seven features from Picard et al. [13] (*N*^+^, *M*_ETC_, *P*^+^, *M*_gly_, *V*, *G* and *R*_max_) and has 12 adjustable parameters (as discussed later, this is fewer than the number required for seven linear models with offsets), a table of which is shown in Supplementary Table S1. In writing down this phenomenological model, we have attempted to account for a physiologically important subset of the data generated in ref. [13], using bottom-up arguments wherever possible. In doing so, many novel, falsifiable, hypotheses are made.

### Parameterizations of a simple biophysical model account for complex observations across range of heteroplasmic load

**Figure 3.**
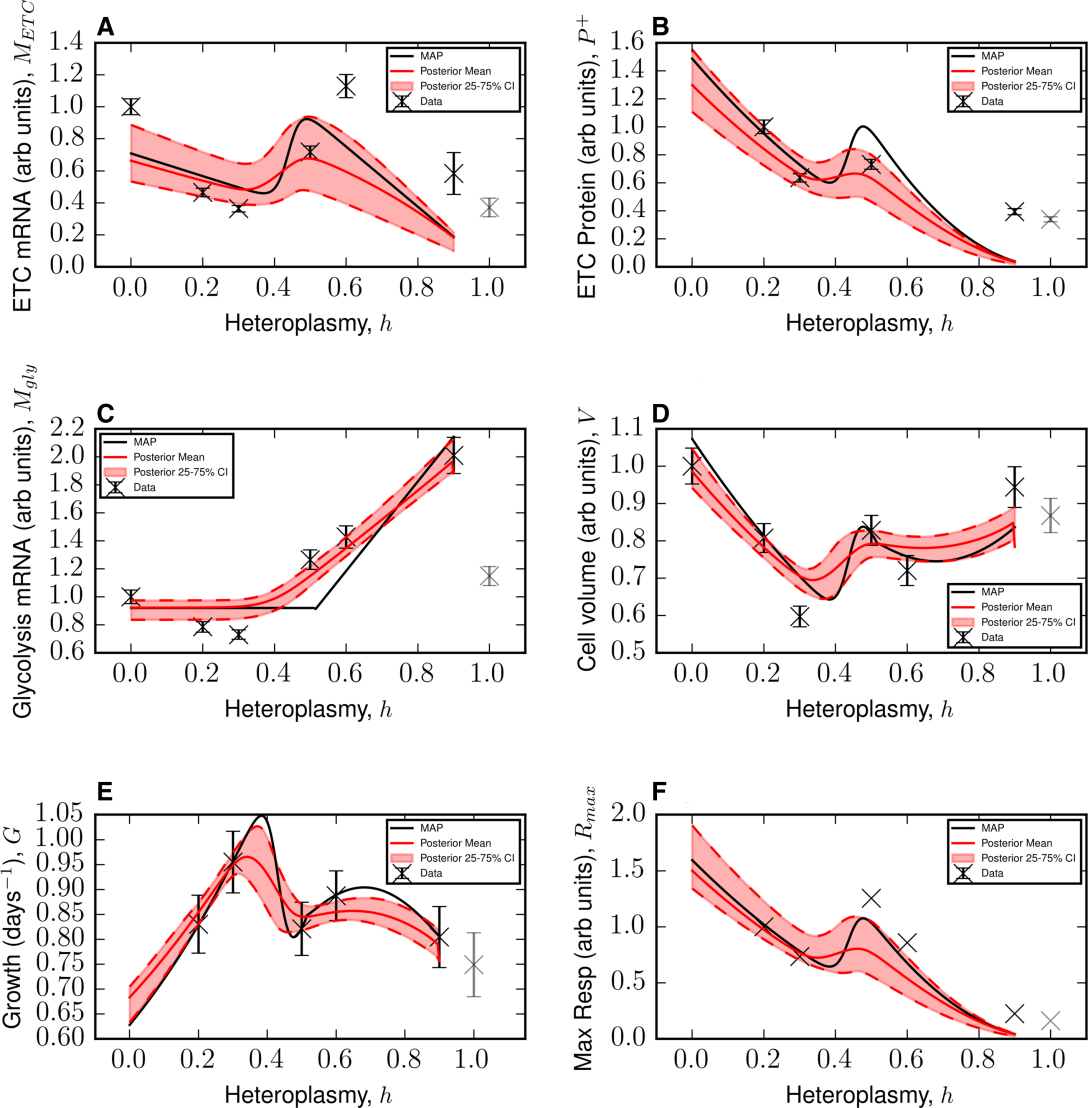
A simple biophysical model is consistent with complex observations across a range of heteroplasmic load. (**A–F**) Approximations for the maximum a posteriori estimate (black line), posterior mean (red line) and 25–75% Bayesian credible intervals (pink bands), for the model fits to selected data from ref. [13]. The model makes predictions over the range 0 ≤ *h* ≤ 0.9 (see the Main Text). Data for *h* = 1 have been plotted in grey, as they have not been used to train the model. Error bars are conservative and merely show the technical variability reported in ref. [13] (see Materials and Methods).

The fit of the model described above is shown in Figure 3. Between 0 ≤ *h* ≤ h*, h* being the critical heteroplasmy where glycolysis is up-regulated (0.34 ≤ h* ≤ 0.44, 25–75% CI), our model reproduces the reduction in ETC transcript pool size. Similarly, we observe that ETC protein pool size also reduces, as does cell volume and maximum respiratory capacity.

Our model successfully captures the transient compensatory responses in ETC mRNA, ETC protein and cell volume which begin around the critical heteroplasmy h*. For heteroplasmies between $h^{*}≲h≲0.5$, ETC mRNA degradation reduces causing ETC mRNA to be up-regulated, along with ETC protein and maximum respiratory capacity. In this region, glycolysis becomes induced above wild-type levels, and cell volume can be observed to also recover.

In excess of *h* ≈ 0.5, our model shows the observed reductions in ETC mRNA, ETC protein and maximum respiratory capacity. We see that continued up-regulation of glycolysis mRNA allows cell volume to remain at an approximately constant value, although diminished relative to a wild-type cell. Consequently, heteroplasmic cells between 0.2 ≤ *h* ≤ 0.9 are predicted to proliferate at a faster rate than wild-type cells (see Figure 3E).

### Key claims and predictions of biophysical model of heteroplasmy

Here, we discuss the interpretations of our model in light of the mathematical description developed above and explore the evidence for the biological insights it provides. We make experimental proposals to validate our claims, which are given in Supplementary Text S4. The set of mechanistic interpretations which follow from our mathematical model are as follows:

- Wild-type mtDNA density is maintained homeostatically at low heteroplasmy.
- There exists a minimum possible cell volume which is approached at the critical heteroplasmy.
- Cells toggle from demand reduction (i.e. cell volume reduction) to supply increase (i.e. glycolysis and ETC mRNA up-regulation), at the critical heteroplasmy.
- Mutant mtDNAs do not significantly contribute to the mitochondrial mRNA pool.
- Mitochondrial tRNAs remain moderately localized to their parent mtDNA.
- Maximum respiratory capacity is determined by ETC protein levels through a linear relationship.
- Cell growth rate is the reciprocal of mean volume, thus smaller cells proliferate faster.

#### Wild-type mtDNA density homeostasis is maintained until a minimum volume is reached near the critical heteroplasmy

**Figure 4.**
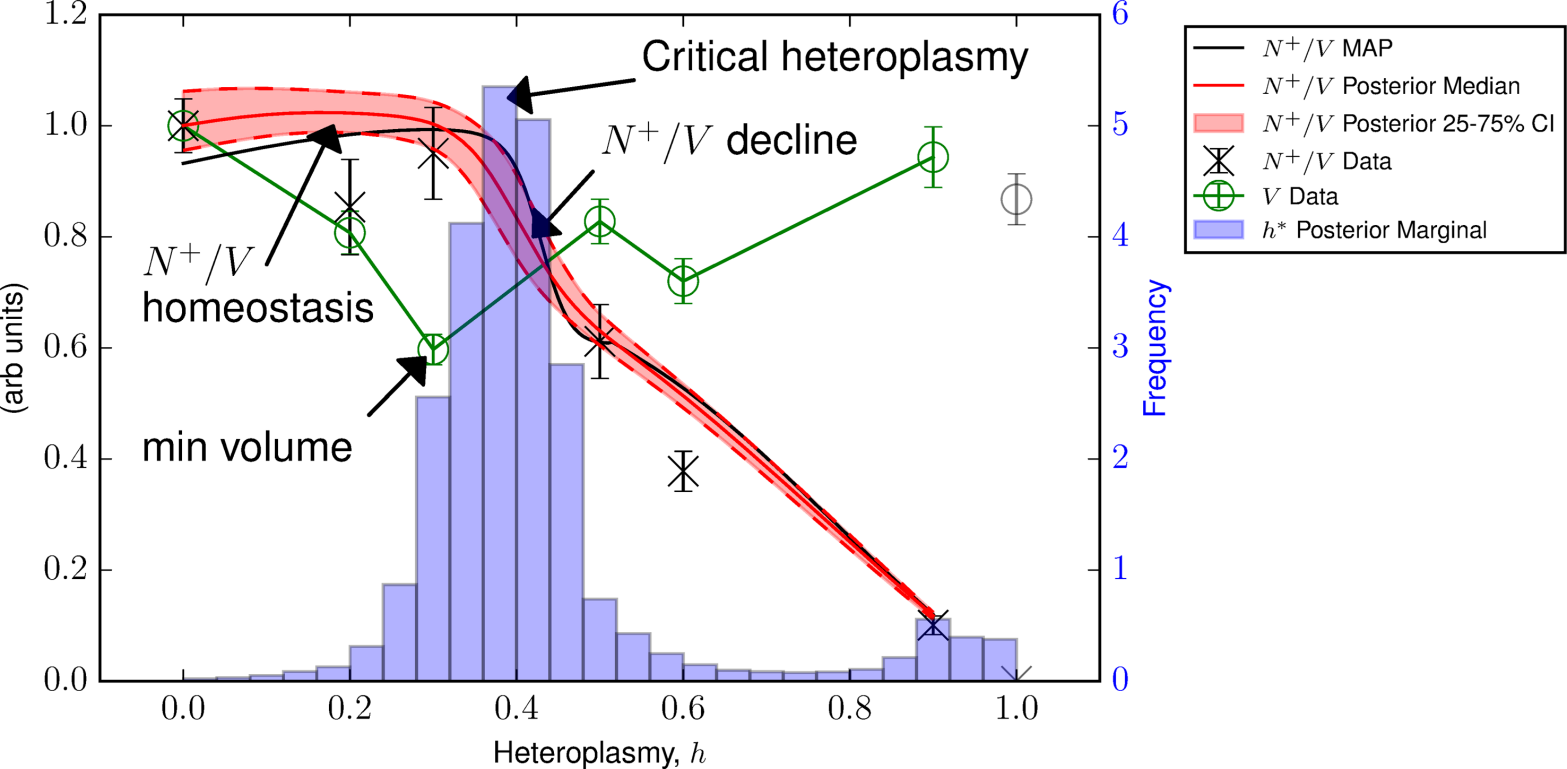
Wild-type mtDNA density (*N*^+^/*V*) homeostasis may trigger supply/demand toggle. Posterior statistics show an initial maintenance of *N*^+^/*V*. When cell volume (*V*) takes its minimum value, the most probable value of h* shortly follows. *N*^+^/*V* then reduces. We suggest that the inability of the cell to maintain *N*^+^/*V* = const, due to the existence of a minimum cell volume, causes cells to toggle in their strategy at h*, from demand reduction to supply increase. Data from ref. [13].

The parameter h* determines the extent of mutant load, for which the cell begins to up-regulate ETC mRNA and glycolysis mRNA. But what causes this change in behaviour, at this particular value of heteroplasmy? By examining the posteriors of our model fit (Figure 3), we infer that cell volume takes its minimum value shortly before the most probable value of h* (see Figure 4). We hypothesize that an attempt to conserve wild-type mtDNA density (*N*^+^/*V*) determines the position of h*.

For $h≲h^{*}$, wild-type mtDNA density is maintained despite increasing heteroplasmy, because cell volume diminishes. As a result of this reduced demand, the cell can tolerate diminished mitochondrial power supply. However, cell shrinkage cannot continue indefinitely and we hypothesize that the cell reaches a minimum cell volume at *h* ≈ h*. Once heteroplasmy exceeds this value, the cell toggles its energy balance strategy from power demand reduction to supply increase, and the cell recovers in volume. We note that this observation was robust to removal of *h* = 0.6 data points where present, see Supplementary Figure S3.

There is evidence in the literature that wild-type mtDNA density is an important quantity. Bentlage and Attardi [17] observed that long-term culture of heteroplasmic MELAS cells resulted in an increase in mtDNA copy number, resulting in increased oxygen consumption. While this was often accompanied by a decrease in heteroplasmy, some cell lines also exhibited this at constant heteroplasmy. This is consistent with the cell attempting to increase the absolute number of wild-type mtDNAs, perhaps to compensate for heteroplasmic load, and suggests that the absolute value of *N*^+^ is a physiologically important quantity.

The density of mitochondrial content per unit cytoplasmic volume has been observed by many authors to be tightly regulated and physiologically predictive. The historical observations of Posakony et al. [18] showed that the mean ratio of mitochondrial content to cytoplasmic volume is kept relatively constant throughout the cell cycle in HeLa cells, occupying ∼10–11% of cytoplasmic area throughout. Similar observations have been reproduced in more recent studies, in various other systems. Rafelski et al. [19] found in budding yeast that mitochondrial content was proportional to bud size, and that all buds attain the same average ratio regardless of the mother's age or mitochondrial content, suggesting a stable scaling relation. Also, Johnston et al. [20] found that the density of mitochondrial mass was predictive of cell cycle dynamics, indicating that *N*/*V* (*N* =  total number of mtDNAs) is physiologically relevant and potentially linked to cell power supply and growth dynamics. Indeed, Jajoo, Paulsson and co-workers [21] found that the density of mitochondrial DNA tracks the quantity of cytoplasm inherited upon division in wild-type fission yeast. Most recently, re-analysis of a work by Miettinen and Björklund [22] found a scaling relationship between cell volume and mitochondrial mass in a population of normal cells [23]: the assumption of constant mitochondrial density was subsequently found to be explanatory in accounting for the data of Miettinen and Björklund [22] using mathematical modelling [23].

We may speculate as to the interpretation of a minimum possible cell volume. One straightforward interpretation is that a minimum cell volume corresponds to a mechanical constraint: a cell may only become so small, because the machinery required to perform tissue-specific metabolic and structural tasks requires a minimum amount of space. Alternatively, a bioenergetic limit to cell volume may exist. For instance, appreciable power demands which scale with, for example, surface area or are invariant to cell size [24–26], may become large relative to power supply as cells become smaller. This is because cellular power supply (through glycolysis and oxidative phosphorylation) likely scales with cell volume. This mismatch in scaling behaviour between power supply and power demand may place a constraint on the minimum cell size.

#### ETC mRNA degradation diminishes at the critical heteroplasmy contributing to power demand/supply toggle

**Figure 5.**
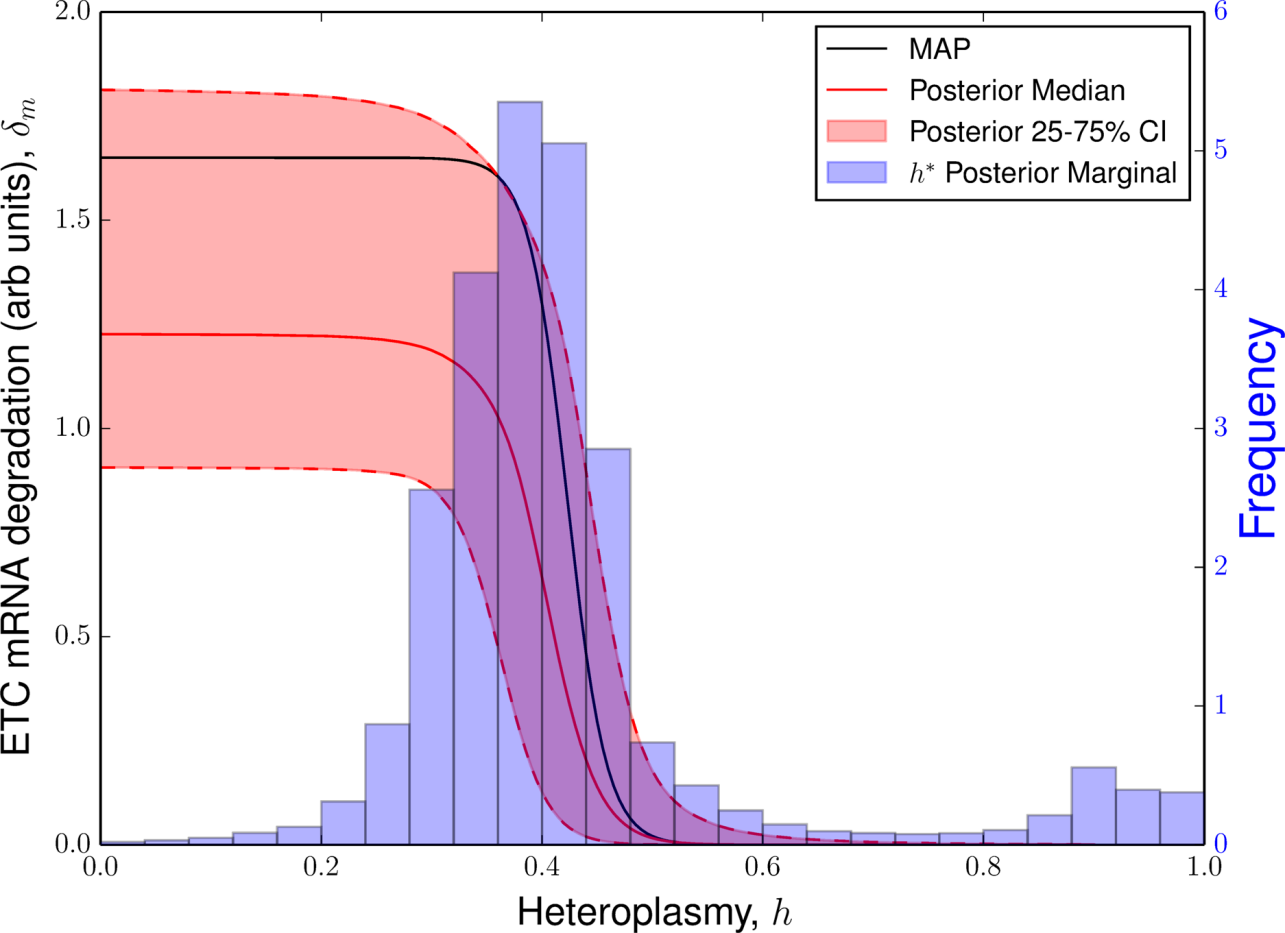
Critical heteroplasmy induces ETC mRNA stabilization. Posterior distributions for ETC mRNA degradation rate and critical heteroplasmy (0.34 ≤ h* ≤ 0.44, 25–75% CI and 0.27 ≤ h* ≤ 0.89, 5–95% CI). It can be seen that the critical heteroplasmy coincides with the reduction in ETC degradation, signalling a power demand/supply toggle.

The induction of glycolysis at the critical heteroplasmy is observed in our model by construction, see eqn (8), since glycolysis is modelled to increase linearly when heteroplasmy exceeds this point. However, by observing the posterior distribution of the ETC mRNA degradation rate (see Figure 5), we see that the critical heteroplasmy also coincides with the beginning of reduction in ETC transcript degradation with respect to heteroplasmy. Since ETC mRNA pool size varies with the inverse of this degradation rate [see eqn (5)], ETC transcripts are consequently up-regulated in tandem with glycolysis transcripts. This occurs until ETC degradation diminishes to negligible levels around *h* ≈ 0.5, where this particular control mechanism becomes exhausted. Thus, the critical heteroplasmy coincides with a shift from power demand reduction to supply increase from both glycolysis and OXPHOS contributions.

Since mtDNA is transcribed as a single polycistronic transcript [27], the relative stoichiometry of individual mRNA species must be controlled via active degradation. This is achieved by a balance between processes which stabilize and degrade mRNA [28]. The Picard dataset can be explored further to seek corroborating evidence, by observing the ratio of ETC mRNA degraders to stabilizers. We find a qualitative similarity between this ratio (see Supplementary Figure S4) and the posterior distribution of the ETC degradation rate (see Figure 5), both displaying a substantial reduction between *h* = 0.3 and *h* = 0.5.

#### Mitochondrial tRNAs are enriched in the vicinity of their corresponding parental mtDNA

The co-location for redox regulation (CoRR) hypothesis states that mtDNA exists in the close proximity of respiratory units for the rapid and direct regulatory control of respiration [29,30]. This hypothesis, which aligns with several leading theories on the origins of complex life [31,32], has recently been supported by studying mitochondrial gene loss throughout the eukaryotic tree of life [33], as well as other experimental evidence reviewed in ref. [30].

Given the CoRR hypothesis, one might expect there to exist a functional link between the genotype of an mtDNA and the phenotype of its spatially closest respiratory units. In other words, mitochondrial nucleoids (protein–DNA complexes which are thought to possess ∼1.1 mtDNA molecules per nucleoid [34]) may each have a limited sphere of influence; this has been put forward as the ‘leaky-link’ hypothesis by other authors [35,36].

The 3243A>G MELAS mutation causes amino acid misincorporations, resulting in unstable translation products [9]. It is reasonable to assume that mutant mtDNAs have a locally higher concentration of mutant tRNAs, since they are the source of these molecules, as well as the presence of cristae which may limit diffusion in the mitochondrial matrix [37]. ETC mRNAs in the vicinity of mutant mtDNAs will more often be translated into unstable protein due to the presence of defective tRNAs. Therefore, mutant mtDNAs may give a weaker contribution to the total functional ETC protein content of the cell than wild-type mtDNAs. Hence, low diffusivity inside the mitochondrial matrix is hypothesized to induce a local phenotype–genotype link [35,36]. We take the limit of mutant mtDNAs providing zero contribution to the total ETC protein content of the cell, rather than a limited contribution, for parsimony — i.e. *P*^+ ^∝^ ^*M*_ETC_*N*^+.^ This is equivalent to assuming a strong local phenotype–genotype link. See Supplementary Text S5 for an alternative model of well-mixed tRNAs.

Evidence in the literature for this claim is mixed. It has been observed that mitochondrial mRNAs localize to mtDNA, suggesting that mtDNA may be a site for mitochondrial translation [38,39]. However, cybrid experiments by Ono et al. [40] involving homoplasmic tRNA mutants 3243A>G and 4269A>G are able to recover their respiratory function by fusing such cells together to form hybrids. Their recovery is presumably due to the diffusion of the healthy form of each tRNA, so that normal proteins may be translated. Yet, one might expect there to exist at least limited local coupling between mtDNA and their gene products under the CoRR hypothesis [29,30,33] and the leaky-link hypothesis [35,36], as described above. It is noteworthy that the cybrid experiments by Ono et al. [40] show recovered respiration after a long adaptation phase of 10 days. Since this is a much larger timescale than that of mtDNA replication, which is typically hours [41], it is conceivable that some kind of long-term adaptation occurred in this system which is still compatible with a strong local phenotype–genotype link; for example, the presence of nucleoids with an mtDNA of each kind of mutant, allowing the local transcription of both of the corresponding wild-type tRNAs. See Supplementary Text S4 and Table S2 for experimental suggestions to determine the extent of tRNA diffusivity.

#### Mutant mtDNAs have a transcriptional defect

It has been hypothesized that, due to the limited diffusion of gene products, mutant mtDNAs may experience a local energy deficiency [36]. This local energy deficiency could conceivably reduce the ability of mutant mtDNAs to perform transcription through a local depletion of ATP. Since the concentration of ATP inside the mitochondrial matrix is low in the physiological setting [42], this may leave processes in the vicinity of mutant mtDNAs with increased susceptibility to local fluctuations in energy availability. If mutated mtDNAs are transcribed more slowly, they may have a lower contribution to the ETC transcript pool.

Alternatively, some other mechanism may generate a functional dependence between mitochondrial transcription and mtDNA genotype. For instance, mitochondrial transcription machinery (such as mitochondrial RNA polymerase, POLRMT) could be preferentially transported to functional mtDNAs, potentially via membrane potential sensing. Since dysfunctional mitochondria are observed to bud off from fused networks [43], it is possible that this mechanism allows the cell to sense the individual performance of mitochondria/mtDNAs [44] and hence target nuclear proteins accordingly. It is also possible that mitochondrial translational machinery could be preferentially targeted to functional mitochondria, which would also result in *P*^+^ ∼ *N*^+^.

We adopted a model of no transcription for mutant mtDNAs, as opposed to limited transcription, for parsimony — i.e. *M*_ETC_ ∼ *N*^+^. See Supplementary Text S5 for an alternative model where mutant mtDNAs provide a non-zero contribution to *M*_ETC_. We provide experimental suggestions for determining the extent of mutant mtDNA transcription in Supplementary Text S4.

#### Cell volume is not explained by cell cycle variations

Our model predicts that cells, on average, change their size as heteroplasmy is varied, due to variation in power supply from OXPHOS and glycolysis. However, since cells vary their volume by a factor of 2 throughout the cell cycle, it is possible that cells with different heteroplasmies spend different durations at various stages of the cell cycle, explaining the observed variation in expected cell volume with heteroplasmy (see Figure 1D). We sought evidence for this hypothesis by computing the ratio of the expression level for genes associated with different stages of the cell cycle [45] (see Supplementary Figure S5). However, we found little evidence to support the enrichment of cell cycle markers at any particular level of heteroplasmy.

#### OXPHOS contributions to power supply are stabilized at the critical heteroplasmy

The relative contribution of OXPHOS to power supply, i.e. *k*_o_*P*^+^/(*k*_o_*P*^+ ^+ *k*_g_*M*_gly_), is also interesting to observe as heteroplasmy is varied. We observed a transient stabilization in OXPHOS contributions around h*. A discussion of this is presented in Supplementary Text S6.

#### Cells proliferate inversely with their size

Owing to our reciprocal model connecting cell volume and growth [see eqn (10)], our model suggests that wild-type cells proliferate more slowly relative to heteroplasmic cells due to their larger size.

#### Maximum respiratory capacity linearly tracks ETC protein content

It has long been suggested that cells above a particular threshold heteroplasmy experience a respiratory defect [10,11,17]. With a more classical interpretation of the threshold effect, we might have expected the need for a model which has switching behaviour in excess of 60% heteroplasmy [10] for maximum respiratory capacity, in analogy with glycolysis transcript levels [see eqn (8)]. However, in our model, we found that a simple linear relationship between ETC protein and maximum respiratory capacity was sufficient to describe the data available [see eqn (11)].

#### Reactive oxygen species may explain the transition to homoplasmy, but the corresponding mode of energy production remains unclear

In eqn (9), we claim that cell volume is determined by the weighted sum of glycolysis transcripts and ETC protein. Over the range 0.9 < *h* ≤ 1, glycolysis transcripts reduce by 57%, whereas ETC protein and cell volume remain comparable, thus breaking the supply = demand relationship, as we have modelled it. Consequently, our model fails to describe the transition from *h* = 0.9 → 1.

A potential explanation for the reduction in glycolysis transcripts over this range comes from the fact that glycolysis provides substrate for oxidative phosphorylation. Damaged ETC proteins may produce an excess of reactive oxygen species (ROS) [46], which can damage mitochondrial proteins, DNA and membranes. If, at high heteroplasmy, any flux through the ETC causes high levels of ROS, then cells may attempt to reduce flux through glycolysis, to avoid production of these species.

Some evidence from Picard et al. supports this hypothesis, where superoxide dismutase (SOD) activity is largely constant with heteroplasmy, except for homoplasmic mutant MELAS cells, which have ∼20% higher SOD activity than wild-type cells (see Supplementary Figure S7D of [13]). Furthermore, it is known that ROS can reversibly inhibit the activity of GAPDH, one of the enzymes involved in glycolysis [47,48].

However, given that fatty acid oxidation (see Supplementary Figure S6) is strongly down-regulated over this range, it remains unexplained how homoplasmic mutant cells maintain their cell volume (and growth rate), given their reduced reliance upon mitochondrial and glycolytic metabolism. Further metabolomic measurements may be required to uncover this mode of energy production.

ATP levels are also observed to decrease over this range (see Supplementary Figure S6E of [13]), which may even suggest that an alternative fuel currency besides ATP supports the growth and size of these cells. However, more careful investigation of this observation may be of value, since it is important to draw the distinction between ATP pool sizes and ATP fluxes, the latter perhaps being more indicative of ATP usage, and the former being indicative of only relative production/consumption rates.

## Discussion

In the present study, through the use of a distilled subset of data from ref. [13] and using minimal arguments, we have attempted to explore the apparent marked difference between the complex multiphasic observations of Picard et al. and the classical step-like models associated with the threshold effect.

We note that the model presented here relies on data from cybrid cells [13]. Such cell lines are often unstable in the number of chromosomes that they possess, and the cybridization process itself may induce transcriptional [49] and metabolic changes [50]. A technical concern one may raise with the dataset of Picard et al. [13] is, therefore, the extent to which their observations may be explained by random genetic drift in the nucleus. However, Picard et al. [13] point out that the observations made with respect to heteroplasmy (at least for a subset of features) appear to be non-random with respect to increasing heteroplasmy: for example, the expression of heat shock proteins appeared to increase monotonically with heteroplasmy, which would not be expected under unbiased random perturbations at each level of heteroplasmy. We note also that cybrid systems are not without merit, as cells have the same nuclear background (although this background may be volatile), and have furthered progress in understanding mitochondrial dysfunction (reviewed in ref. [51]). As is the case generally with model biological systems, it is important to seek corroborating evidence from multiple model systems before accepting the observations from any single experiment. Hence, we have suggested a range of experiments to validate the hypotheses we have generated here.

We have argued that a single critical heteroplasmy, h*, is sufficient to explain this subset of data over the heteroplasmy range 0 ≤ *h* ≤ 0.9 and that other multiphasic behaviour arises naturally from the simple physical/biological assumptions of our model. Our model suggests that cells undergo a power demand/supply toggle at h*, from demand reduction to supply increase. We hypothesize that homeostasis in wild-type mtDNA density is maintained via cell volume reduction, ensuring that the available functioning power sources are matched to a corresponding level of cellular demand, until a minimum cell volume is reached which coincides with h*. This triggers the demand/supply bioenergetic toggle where energy production pathways are up-regulated. We believe that this re-emphasizes the need for quantification of single-cell mtDNA content to be associated with volume measurements of the same cell: mtDNA density is a relevant physiological variable [18–21,52]. We find that the mode of energy production over the range 0.9 ≤ *h* ≤ 1 is unclear, and that further metabolomic investigations may be required to determine this.

Our model further predicts that mutant mtDNAs have a reduced contribution to transcription and that tRNAs have low diffusivity. These hypotheses potentially follow from the ‘leaky-link’ hypothesis whereby mtDNAs have a limited extent of influence, such that there exists a phenotype–genotype link between an mtDNA and its spatially closest respiratory units [35,36]. We also predict that a relationship exists between mean cell volume and cell growth.

A potential consequence of our predictions is that modulation of either mtDNA copy number, wild-type mtDNA copy number or wild-type mtDNA copy number density to ensure optimal values of wild-type mtDNA copy number density could be valuable control axes in therapy. Increasing mitochondrial DNA copy number, for instance through activation of the PGC-1α pathway, may facilitate the increase in cell volume, deferring the critical heteroplasmy to higher values by delaying the approach towards a minimum cell volume. We might reason that this enhances a wild-type phenotype at higher heteroplasmy values, potentially deferring the full MELAS phenotype to higher heteroplasmies, which typically appears between ∼50 and 90% mutant load [53]. Indeed, it has been found that increasing mitochondrial biogenesis can ameliorate mitochondrial myopathy *in vivo* [54].

We might also argue that as cells toggle from power demand reduction to supply increase, further bolstering of this compensatory response may have clinical significance. For instance, since we observe that cells switch to glycolytic metabolism to compensate for diminishing mitochondrial power supply, further encouragement of this energy mode may be therapeutic. This is supported by the recent observation that promoting the hypoxia response is protective against multiple forms of respiratory chain inhibition [55]. Alternatively, since we predict that cells innately down-regulate ETC mRNA degradation, seeking to up-regulate mitochondrial transcription may aid the cell in maintaining a sufficient mRNA pool size. Furthermore, promoting alternative energy production pathways such as fatty acid oxidation via the ketogenic diet may also aid in reducing the dependence on oxidative phosphorylation. This diet has been associated with increased mitochondrial transcripts [56], mitochondrial content [56,57] and has been shown to slow mitochondrial myopathy progression in transgenic Deletor mice [57]. Indeed, the diet has recently been used in clinic as an adjunctive therapy for a patient suffering from MELAS, harbouring the 3260A>G mutation, which successfully decreased the frequency of seizures and stroke-like episodes [58].

## Abbreviations

CoRR: co-location for redox regulation
ETC: electron transport chain
MELAS: mitochondrial encephalomyopathy, lactic acidosis and stroke-like episodes
OXPHOS: oxidative phosphorylation
ROS: reactive oxygen species
SOD: superoxide dismutase
