# Supplementary material for: Mitochondrial DNA density homeostasis accounts for a threshold effect in a cybrid model of a human mitochondrial disease
Source: Biochem J. 2017 Nov 24;474(23):4019–34. doi: 10.1042/BCJ20170651 (PMC5705840; doi:10.1042/BCJ20170651)
Supplement: Supplementary Figures and Tables [file BCJ-474-4019-s1.pdf]

# Supplementary Information

## Text S1

### Transformation to Per-Cell Dimensions using Cell Volume

In this section we show that it is necessary to multiply measurements of protein and mRNA levels, when determined by Western blot and RNA-seq respectively, by cell volume to transform the data to mean cellular measurements.

Consider a Western blot experiment determining the levels of a gene (gene $i$) in two conditions (A and B). Denote the number of proteins per cell of gene $i$, as $n_i^A$, where the superscript denotes condition A. Let us also denote the total number of proteins per cell as $N^A$.

When one performs a Western blot, the protein of interest is stained with an antibody, cells are lysed, and a sample of fixed protein mass ($m$) is taken from the lysate. If we denote the number of proteins for the gene of interest in the sample as $P_i^A$, then we may write

$$P_i^A = \frac{n_i^A}{N^A} m, \tag{S1}$$

since the proportion of protein $i$ in the sample is determined by the proportion of protein $i$ in the proteome ($n_i^A/N^A$). Western blot experiments also tend to be normalised by a loading control ($c$), so the normalised measurement we have access to is

$$\frac{P_i^A}{P_c^A} = \frac{n_i^A}{n_c^A} \tag{S2}$$

which corresponds to the data given in Picard et al. [13].

Now, consider a perturbation in condition B, causing the amount of protein for gene $i$ to be $n_i^B$, and the mean cell volume to experience a fold-change $V_f$, as in Figure S12. Consequently, $N^B = V_f N^A$, since total protein content scales with the volume of the cell. Using the reasonable assumption that the loading control is a gene whose expression also scales with cell volume (e.g. $\beta$-actin, as in Picard et al. [13]), then $n_c^B = V_f n_c^A$. It follows that

$$\frac{P_i^B}{P_c^B} = \frac{n_i^B}{V_f n_c^A}. \tag{S3}$$

Then, if we are interested in the relative fold-change expression of the protein between the two conditions, then we take the ratio

$$\frac{P_i^B/P_c^B}{P_i^A/P_c^A} = \frac{n_i^B/n_c^A}{n_i^A/n_c^A} \cdot \frac{1}{V_f} = \frac{n_i^B}{n_i^A} \cdot \frac{1}{V_f} \tag{S4}$$

Thus, the quantity on the left hand side of Eq. (S4), which is what one usually measures in a Western blot, has a multiplicative-bias of $1/V_f$. Therefore, if one is interested in per-cell protein changes, the appropriate quantity of interest is

$$\frac{P_i^B/P_c^B}{P_i^A/P_c^A} \cdot V_f. \tag{S5}$$

Hence, we multiply each protein measurement by $V(h) \equiv V_f(h) = \langle V_k(h)\rangle_k / \langle V_k(h=0)\rangle_k$, where $\langle \ldots \rangle_k$ denotes the sample mean over technical replicates $k$, such that $V(0) = 1$.

A similar argument applies to RNA-seq data, since a fixed mass of mRNA is extracted for an RNA-seq experiment, so an analogous pair of equations to Eq.(S1) in conditions A and B holds. Using the assumption that $N^B = V_f N^A$, and denoting the number of mRNA molecules in each sample with $M$, it can be shown that

$$\frac{M_i^B}{M_i^A} = \frac{n_i^B}{n_i^A} \cdot \frac{1}{V_f}. \tag{S6}$$

## Text S2

### Generative Model Description

We used a Bayesian framework to find the supported parameter values given the data, using the Metropolis-Hastings algorithm [1]. To do this, we included an additional 6 noise parameters, for the features where parameter inference was performed (i.e. all of the features except $N^+$, which has no free parameters, see

Eq.(4)). For these 6 features ($M_{\text{ETC}}, P^+, M_{\text{gly}}, V, G, R_{\max}$), we assumed that the data were generated subject to Gaussian noise.

Thus, the full statistical model contains 12 parameters (excluding 6 noise parameters for each feature), with 32 data points which enter the likelihood (after excluding $h = 1$ data). To summarise, counting the 6 features which have free parameters, the model consists of $12/6 = 2$ mean parameters per feature, on average. Note that simply fitting linear models to the 6 features in Fig. 1 would also require 2 parameters per feature. The model fit is shown in Fig. 3.

To connect our model of mean cellular behaviour $\mathcal{S} = \{M_{\text{ETC}}, P^+, M_{\text{gly}}, V, G, R_{\max}\}$, to the data of Picard *et al.* [13], we assume that the sample mean of feature $i$ ($y_{i,j}$) at a discrete value of heteroplasmy $h = j$ is generated via Gaussian noise ($\mathcal{N}(\mu, \sigma)$) whose mean corresponds to one of the models $\mathcal{S}$,

$$y_{i,j} = \mathcal{M}_i(h) + \mathcal{N}(0, \sigma_i), \tag{S7}$$

where $\mathcal{M}_i(h)$ is an element from the set of models $\mathcal{S}$. We stress that the data we train our model on, $y_{i,j}$, is the *sample mean*, rather than the raw data. This is a less common approach; however, we believe that it is appropriate as individual replicates only give us information on the technical variability measured in [13], whereas the total error is a combination of both technical and biological variability. Training our models on individual replicates would be likely to underestimate the true variability of the data, so we favoured training on the sample mean only. This raises the challenge of establishing an appropriately permissive model for our uncertainty in $\sigma_i$.

We can infer the distribution of the parameters ($\theta$) of the models $\mathcal{S}$, given the data $y_{i,j}$, using Bayes rule and a prior distribution over $\theta$ ($P(\theta)$)

$$P(\theta|y_{i,j}) = \frac{P(y_{i,j}|\theta)P(\theta)}{P(y_{i,j})}. \tag{S8}$$

The log-likelihood in this case is

$$\log[P(y_{i,j}|\theta)] = \sum_{i,j} -1/2\log(2\pi) - \log(\sigma_i) - \frac{1}{2}\left(\frac{y_{i,j} - \mathcal{M}_i(j)}{\sigma_i}\right)^2. \tag{S9}$$

We drop the constant $\sum_{i,j} -1/2\log(2\pi)$ from our log-likelihood, since we will only be interested in differences in the log-likelihood to perform Bayesian inference using the Metropolis-Hastings algorithm [1].

We used exponential priors $\sigma_i$

$$P(\sigma_i) \propto \exp(-\lambda_i \sigma_i) \tag{S10}$$

as our error model. The constant $\lambda_i$ was chosen such that the scale of decay of probability was on the same scale as the range of the data. Noting that $\langle P(\sigma_i)\rangle = 1/\lambda_j$, we chose

$$\lambda_i = \frac{\Omega}{\max_j\{\hat{y}_{i,j}\} - \min_j\{\hat{y}_{i,j}\}} \tag{S11}$$

where $\Omega$ is a hyper-parameter of the prior and $\Omega \geq 0$. Note that we may interpret $\Omega = 0$ as an improper uniform prior, since $P(\sigma_i) = \text{const}$ in this case.

We began with $\Omega = 0$ as the most permissive choice of prior possible, given the model in Eq.(S10). We found that when $\Omega = 0$ *the maximum a posteriori estimates were qualitatively similar* to choosing $\Omega = 2$ (our final choice which we justify below) see Fig. 3 ($\Omega = 2$) and Figure S13A-F ($\Omega = 0$). However, we found that the posterior 25-75% credible interval supported model fits for $M_{\text{ETC}}$, $P^+$ and $R_{\max}$ which were relatively poor when $\Omega = 0$, compared to $\Omega = 2$ (see Figure S13A-F). We determined that large values of $h^*$ were indicative of purely linear fits to the data, which is unlikely given the wider body of evidence demonstrating the nonlinearity of the threshold effect. This is seen in Figure S13G-L (high $h^*$, poorer fit) when compared with Figure S13M-R (low $h^*$, better fit). Comparison between Figure S13G and Figure S13M is particularly noteworthy, where the 25-75% posterior credible interval for high $h^*$ sub-samples predicts $M_{\text{ETC}} \approx 0$ for all values of $h$, which is physiologically implausible, whereas low $h^*$ sub-samples display non-linear fits which more faithfully track the data. Figure S14 shows that the high $h^*$ mode is of comparable prevalence to the low $h^*$ mode when $\Omega = 0$.

We therefore investigated the sensitivity to choice in $\Omega$ in Figure S14. We see that increasing $\Omega$ reduces the width of the marginal posterior distribution of $h^*$, constraining the posterior distribution to lie around

the nonlinear solutions shown in Fig. 3. We found that the permissive prior $\Omega = 2$ was sufficient to strongly subdue this, physiologically implausible, large $h^*$ mode. This can be interpreted as a prior belief that our model uncertainty is, on average, 50% of the range of the data (since $\langle P(\sigma_i)\rangle = \lambda_i^{-1}$). We believe this to be a sensible prior choice, encoding our prior belief that the threshold effect is nonlinear while providing only a gentle constraint on parameters.

We favoured uniform priors on the remaining parameters so that the posterior would be dominated by the likelihood. However, a number of the parameters in the model were uncertain over orders of magnitude; in these cases, we allowed the log of these parameters to take uniform distributions. Explicitly, our priors were chosen as:

$$P(h^*) = \text{unif}(0, 1) \tag{S12}$$
$$P(\ln(k_{\text{mRNA}})) = \text{unif}(-10\ln 10, 2\ln 10) \tag{S13}$$
$$P(f_m) = \text{unif}(0, 1) \tag{S14}$$
$$P(\ln(k_m)) = \text{unif}(-10\ln 10, 2\ln 10) \tag{S15}$$
$$P(\ln(\delta_p)) = \text{unif}(-10\ln 10, 2\ln 10) \tag{S16}$$
$$P(\ln(k_o)) = \text{unif}(-10\ln 10, 2\ln 10) \tag{S17}$$
$$P(\ln(k_g)) = \text{unif}(-10\ln 10, 2\ln 10) \tag{S18}$$
$$P(c_1) = \text{unif}(-10, 10) \tag{S19}$$
$$P(m_2) = \text{unif}(0, 100) \tag{S20}$$
$$P(k_{gr}) = \text{unif}(0, 100) \tag{S21}$$
$$P(\ln(k_p)) = \text{unif}(-10\ln 10, 2\ln 10). \tag{S22}$$

The ranges for $h^*$ and $f_m$ are justified since these quantities can physically only be between 0 and 1. $c_1$ and $m_2$ are parameters of linear models for $M_{gly}$ (see Table S1 and Eq.(8)) for data which has been normalized to the scale of 1; therefore priors were chosen with suitably large ranges. Similarly for $k_{gr}$, a proportionality constant relating growth to cell volume ((see Table S1 and Eq.(10)), we expect $k_{gr}$ to be of the order of 1, since the data has been normalized, and chose suitably relaxed priors. The ranges for all other parameters, which were sampled in log-space due to our greater uncertainty of their values, were chosen to be suitably large as to be unlikely to reach the boundary of the prior during sampling with MCMC.

The parameters $\beta$ and $k_{\text{mRNA}}$ from Eq.(5) were highly correlated. For more efficient chain mixing, we rearranged Eq.(5) into the form

$$M_{\text{ETC}} = \frac{\zeta}{\frac{1}{k_{\text{mRNA}}} + \frac{1}{1+\exp[k_m(h-h_0)]}}, \tag{S23}$$

where $\zeta = \beta/k_{\text{mRNA}}$, and used the prior

$$P(\ln(\zeta)) \sim \text{unif}(-10\ln 10, 2\ln 10) \tag{S24}$$

such that the boundaries for the uniform prior were relatively relaxed.

We performed the Metropolis-Hastings algorithm [1] to sample from the posterior, using a Gaussian random walk as our transition kernel, whose covariance matrix was determined from a trial run of the adaptive Metropolis algorithm [3]. All code was written in either Python or C, and is available upon request. The MCMC chain trajectory is presented in Figure S7.

## Text S3

### Justification of ETC mRNA and protein

Consider a *single* molecule of wild-type mtDNA which, when transcribed, generates mRNA for the electron transport chain (ETC), which we denote as $m_{ETC}$. Transcripts are generated according to a deterministic process (stochasticity in gene expression [4] is neglected in this picture) with rate ($\beta$) and also passively degrade at some basal rate ($\delta_m^b$). We consider a controlled, active degradation process ($\delta_m^a$) that acts in addition to the background level. Thus, at the single mtDNA level, we may write down the differential equation

$$\frac{\mathrm{d}m_{\text{ETC}}}{\mathrm{d}t} = \beta - \left(\delta_m^a + \delta_m^b\right) m_{\text{ETC}}, \tag{S25}$$

where we assume that $\beta, \delta_m^b$ are constant.

Control of the expression levels of different mitochondrial genes is manifest at the level of mRNA degradation [28], because mtDNA is transcribed as a single polycistronic transcript [27]. We therefore use the simplifying assumption that, in the pathogenic case, mitochondrial mRNA is also controlled at the level of degradation. Thus we allow the active degradation to vary with heteroplasmy $\delta_m^a = \delta_m^a(h)$, and assume the transcription rate to be constant.

Cells are measured at steady-state, so setting the derivative to zero yields

$$m_{\mathrm{ETC}} = \frac{\beta}{\delta_m^a + 1}, \tag{S26}$$

where $\delta_m^b$ has been absorbed into the definitions of $\beta$ and $\delta_m^a(h)$. Assuming $N^+$ scaling, whereby only wild-type mtDNAs contribute to the transcript pool, we arrive at an expression for *cellular* levels of ETC mRNA:

$$M_{\mathrm{ETC}} = \frac{\beta}{\delta_m^a + 1} N^+.$$

Dropping the $a$ superscript yields Eq.(5).

Note that, in our Bayesian inference, we chose to express the constant $h_0$ in Eq.(6) in terms of the critical heteroplasmy $h^*$ using the expression $h_0 = h^* - \ln[(1-f_m)/f_m]/k_m$, where $0 < f_m \leq 1$. This simply expresses the location of $h^*$ in terms of the fraction $f_m$ of the sigmoid's maximal value. Intuitively, if $f_m$ and $k_m$ are sufficiently large, $h^*$ signals the beginning of reduction in ETC degradation.

For ETC protein, we assume that the following equation holds at the cellular level

$$\frac{\mathrm{d}P^+}{\mathrm{d}t} = \lambda M_{\mathrm{ETC}} N^+ - \delta_p^b P^+ \tag{S27}$$

where $\lambda = \text{const}$, and we assume there is no active degradation of ETC protein. At steady-state

$$P^+ = \frac{M_{\mathrm{ETC}} N^+}{\delta_p^b} \tag{S28}$$

where $\lambda$ is absorbed into the definition of $\delta_p^b$. Dropping the $b$ superscript yields Eq. (7).

### Justification of cell volume scaling for power demand

A reasonable general model for the way in which power demands of a mammalian cell scale with its volume ($V$) is $k_1 V + k_2 V^{2/3} + K + f(N, V)$, where $k_i$ are proportionality constants. Each term may be interpreted as: $k_1 V$ are demands which scale with cell volume; $k_2 V^{2/3}$ scale with cell surface area; $K$ are demands which are constant for a cell (for example, the cost of replicating the genome); and $f(N, V)$ is an unknown function corresponding to proton leak, which potentially depends upon mitochondrial mass (or alternatively mtDNA copy number, $N$) and cell volume. These are the dominant power demands of mammalian cells, as determined by [24–26].

Many energy-consuming processes in mammalian cells directly depend upon cell size; for example, a model system used by Buttgereit *et al.* found that ∼30% of oxygen consumption corresponded to plasma membrane transporters, and ∼20% corresponded to protein synthesis [24]. We assume that protein synthesis scales proportionally with $V$, because 60% of total cellular dry mass is protein [10] and mass scales with volume. Also, we may assume that plasma membrane power consumption scales with cell surface area, which scales with $V^{2/3}$. By using volume and surface area contributions alone, we may account for ∼50% of the power demands of the cell, which is 63% of the accountable power demands for this model system since only 80% of total respiration rate could be attributed to particular processes in their study [24].

Thus, assuming that all power consumption is due to surface area or volume contributions then, using the data of [24], a reasonable model for power demand might be $f(V) = 0.4V + 0.6V^{2/3}$, the volume parameter being $20/(20+30) = 0.4$. However, we see in Fig. 1D that the normalized volume data lie in the range $0.6 \lesssim V \lesssim 1$. In this region, the functions $f(V)$ and $g(V) = V$ are similar, with a difference of no more than ∼7%. Thus, $g(V) = V$ is a reasonable approximation for total power demands in this case.

We note, however, that the above proportions depend on the environment of the cell [24, 25] as well as the tissue type (reviewed in [26]), often showing variation on the order of tens of percent. In light of this uncertainty, and for the sake of parsimony, we make the simplifying assumption that total power demand scales purely with cell volume, see Eq.(9).

### Expected cell volume and growth rate

Two of the simplest models for how cells may grow throughout the cell cycle are linear and exponential growth. We show below that a relationship exists between growth rate and the mean cell volume in an asynchronous population of cells under a linear model. Furthermore, assuming an exponential model, growth rate and mean cell volume are independent.

Firstly, we assume that the number of cells obey a pure-birth process, in other words the death rate of cells is negligible. If the initial number of cells ($N_0$) is large, then we can use a deterministic model of cell growth, $N(t) = N_0 \exp(Gt)$, where $N(t)$ is the number of cells at time $t$ and $G$ is the growth rate of cells, as described in Eq.(10). Assuming that the number of cells doubles every cell cycle period ($t_d$), then

$$G = \frac{\ln(2)}{t_d}. \tag{S29}$$

Under a linear model of cytoplasmic growth through the cell cycle, the volume of an individual cell may be written as

$$V_c(t) = V_0 + \lambda t \tag{S30}$$

where $V_c$ is the volume of an individual cell, $V_0$ is the volume of a cell just after division (assumed to be constant for all cells) and $\lambda = V_0/t_d = \text{const}$ is the cytoplasmic growth rate. We note that, by our assumption of $\lambda = \text{const}$ and a linear growth model, smaller cells require less time to double in volume and consequently will proliferate faster (as shown below). From an energetics perspective, a simple model is that cellular power demand relating to proliferation scales with $\lambda$, i.e. more energy per unit time is required to increase the cytoplasmic growth rate. Since we model $\lambda = \text{const}$, we expect power demands associated with proliferation to also be constant regardless of the volume of the cell (and hence independent of heteroplasmy in the model presented in the Main Text). We use the parsimonious ansatz that this power demand is small relative to the power demands of maintaining cytoplasmic volume, and hence we neglect a $\lambda$-dependent term in Eq.(6).

In an asynchronous population, we assume that each cell is distributed uniformly through the cell cycle, in other words

$$T \sim \text{unif}(0, t_d) \tag{S31}$$

where $T$ is a random variable describing the position in time, of a cell in its cell cycle.

We wish to find the expected value of cell volume, ($\mathbb{E}(V_c) \equiv V$, as described in Eq.(9)), given the assumption of Eq.(S31). Eq.(S30) can be viewed as a transformation of the random variable $T$. If $X$ is a continuous random variable, then for any transformation $Y = r(X)$, $\mathbb{E}(Y) = \int r(x)P(x)\mathrm{d}x$, where $P(x)$ is the probability distribution corresponding to the random variable $X$ [11]. This implies that $\mathbb{E}(V_c) = 1/t_d \int_0^{t_d} V_c(t)\mathrm{d}t$. For Eq.(S30), this yields $\mathbb{E}(V_c) = 3V_0/2$, but since $V_0 = \lambda t_d$, then Eq.(S29) yields

$$G = \frac{3\lambda \ln(2)}{2\mathbb{E}(V)}, \tag{S32}$$

i.e. $1/G \propto \mathbb{E}(V) \equiv V$.

If, however, we assume an exponential model of cell growth through the cell cycle

$$V_c(t) = V_0 \exp(\gamma t) \tag{S33}$$

then $t_d = \ln(2)/\gamma$ and $\mathbb{E}(V_c) = V_0/\ln(2)$ which cannot be written in terms of $t_d$ and therefore $G$ is independent of $V$.

The above makes intuitive sense: if a cell grows linearly, then a larger cell will need more time to double in size than a smaller cell, if their growth rates are the same. On the other hand, if a cell grows exponentially, then regardless of its initial size, the doubling time is constant, given a fixed cytoplasmic growth rate.

Since there is presumably a wide class of cell growth dynamics where cell size is dependent on growth rate, we favoured a linear model for its simplicity. Measurements by Tzur *et al.* show that, on average, under both a linear and exponential model of cytoplasmic volume growth, the rate constant varies with time [12]. However, the implication of this for the relationship between $V$ and $G$ remains unclear.

## Text S4

Below, we will propose potential experiments to test the corresponding claims made in Key Claims and Predictions of Biophysical Model of Heteroplasmy.

### Wild-type mtDNA density homeostasis is maintained until a minimum volume is reached at the critical heteroplasmy

If $N^+/V$ is a quantity kept under homeostasis, then under wild-type conditions, perturbations to mtDNA copy number may be expected to cause changes in cell volume. This might be testable by reducing mtDNA copy number with chemicals such as ddC, or increasing it through PGC-1$\alpha$ overexpression, which, in the absence of other homeostatic effects, we expect to reduce and increase mean cell volume respectively.

A second testable prediction is that a minimum cell volume ($V_{\min}$) causes bioenergetic toggling at $h^*$. We should be aware of the two potential interpretations of $V_{\min}$ raised: (1) bioenergetic and (2) mechanical. If $V_{\min}$ is bioenergetic, then raising the power demands of the cell which do not scale with volume, may induce $h^*$ to be encountered earlier. This could be achieved by increasing the amount of DNA in the nucleus which must be replicated, creating a one-time cost to the cell per cell cycle. This is not expected to affect any mechanical constraints since DNA content is not directly indicative of nuclear size [13]. Ideally, the amount of DNA introduced should be large (i.e. billions of base pairs), be replicated, and not interfere with normal functioning of the nucleus. This could be achieved by chemically inducing polyploidy, for instance by using Noscapine [14]. The location of $h^*$ could again be determined by performing RNA-seq, and observing the upregulation of glycolysis with heteroplasmy.

If increasing the power demands of the nucleus yields no change in the distribution of $h^*$, then a mechanical constraint could be more relevant. This could be tested by perturbing cell volume. Reducing cell volume under this hypothesis is expected to shift $h^*$ to lower values of heteroplasmy, which could be determined via RNA-seq.

### Mitochondrial tRNAs are enriched in the vicinity of their corresponding parental mtDNA

The spatial distribution of mitochondrial tRNAs relative to mtDNA would be most directly determined by fluorescent labelling of mitochondrial tRNA and mtDNA. MtDNA labelling could be achieved through picoGreen staining [15]. Labelling of processed tRNAs within mitochondria is more difficult, but methods exist for labelling mRNA in both fixed cells [16] and dynamically [18, 39] within mitochondria, which may be informative. The experimental suggestion put forward Busch *et al.* [36] to probe the strength of the genotype-phenotype link, via super-resolution microscopy [20], by determining the existence of focal deficiencies in ETC protein concentration would also be illuminating.

### Mutant mtDNAs have a transcriptional defect

If mutant mtDNAs have a transcription defect, then the abundance of mRNA encoding mutant tRNAs relative to mRNA encoding wild-type tRNAs would be expected to be smaller than $h$. This measurement could be performed by RT-qPCR, with probes which target the single-nucleotide polymorphism associated with the 3243A>G mutation, to probe the ratio of mRNA derived from mutant and wild-type mtDNAs. Superresolution microscopy to determine the existence of focal deficiencies in ETC protein would also potentially indicate the existence of a local genotype-phenotype link, which would support this hypothesis [20, 36].

### Cell volume is not explained by cell cycle variations

To separate the potential confounding influence of the cell cycle on mean cell size, heteroplasmic cells could be transfected with Fucci markers [21], and relative enrichment of cell cycle stages determined.

### Cells proliferate inversely with their size

To determine the dependence of growth rate on mean cell volume, wild-type cells could be synchronised, and sorted by their volume. These cells could then be plated and released from synchronisation, and the growth rate of cells measured similar to that described in Materials and Methods. Synchronisation is necessary, because cell volume is expected to vary by a factor of 2 through the cell cycle, so any sorting would otherwise be strongly confounded by the cell cycle. A potential alternative to synchronisation, which can be stressful to cells, is to label genes associated with a particular stage of the cell cycle, and sort based on both this fluorescence signal and cell volume.

### Maximum respiratory capacity linearly tracks ETC protein content

Measurements of maximum respiratory capacity at $h = 0$, as well as measurement of ETC protein levels at $h = 0.6$ and $h = 0$, may help determine whether a simple linear relationship is sufficient, or whether a more complex model is justified.

### Summary

A summary of the experimental proposals outlined are given in Table S2

## Text S5

### Alternative models: Mutant mtDNA and transcription

Eq.(5) states that mutant mtDNAs do not contribute significantly to the transcript pool. We can relax this constraint by replacing Eq.(5) with

$$M_{\text{ETC}} = \frac{\beta}{\delta_m + 1}(N^+ + \mu N^-) \tag{S34}$$

where $0 \leq \mu \leq 1$ and $N^- = hN$, where $N$ is the total number of mtDNAs, which we treat as a constant.

Using the uniform prior

$$P(\mu) = \text{unif}(0, 1), \tag{S35}$$

we sampled from the posterior, as described in Text S2. The MCMC trajectory is shown in Figure S8. The marginal posterior density for $\mu$ in Figure S8, shows that $\mu \approx 1$ is the most likely value of the parameter, in other words mutant mtDNAs contribute equally to the transcript pool, compared to wild-type molecules. However, draws from the posterior distribution of $M_{\text{ETC}}$ were often purely linear and thus inappropriate for understanding threshold effects (see Figure S16). We consequently rejected this model in favour of the model presented in the main text.

### Alternative models: tRNA misincorporation model

Eq.(7) states that ETC protein is generated when ETC mRNA is in contact with wild-type mtDNA, suggesting that tRNAs affected by the MELAS mutation, leucine-UUR, remain local to their parent mtDNAs. The alternative is that mitochondrial tRNAs are well diffused amongst mitochondrial mRNAs. If we assume that a mutant tRNA causes a misincorporation during translation with 100% efficiency, then the number of misincorporations per protein follows a binomial distribution. We assume that the probability of a single misincorporation is $h$. We further assume that proteins have a mutational tolerance of $x$ misincorporations, or less, before they are considered mutated (and consequently degraded). With these assumptions, the expected proportion of mutant proteins ($m_p$) will be

$$m_p = 1 - F(x|N, h) \tag{S36}$$

where $N$ is the number of leucine-UUR residues per protein, and $F(x|N, h) = P(X \leq x)$ is the cumulative distribution function of the binomial distribution, for $N$ trials, $x$ successes, and probability of success $h$. A plot of $m_p$ is given in Figure S11, for different mutational tolerances $x$ against heteroplasmy.

We can therefore use an analogous expression to Eq.(7) for $P^+$, in the case of well-diffused tRNAs

$$P^+ = \frac{M_{\text{ETC}} F(x|N, h)}{\delta_p^b}. \tag{S37}$$

By replacing Eq.(7) with Eq.(S37), we again sampled from the posterior as described in Text S2. We chose $N = 8$, which is the average number of susceptible residues in the 11 mitochondrially-encoded subunits considered (see caption of Fig. 1) [9]. Our prior for the unknown tolerance to misincorporations, $x$, was chosen as a discrete uniform prior

$$P(x) = \begin{cases} \frac{1}{9}, & x = 0, 1, \ldots, 8 \\ 0, & \text{otherwise.} \end{cases} \tag{S38}$$

The MCMC trajectory is shown in Figure S9, and the model fit in Figure S11. Draws from the posterior distribution of $M_{\text{ETC}}$ were often purely linear and thus inappropriate for understanding threshold effects

(see Figure S16). We consequently rejected this model, in favour of the model presented in the main text. Furthermore, observing the marginal posterior distribution of the misincorporation tolerance $x$ in Figure S9, we see that the most likely value of the parameter is $x = N = 8$. In other words, ETC proteins are immune to the MELAS mutation, which we believe to be incorrect [9]. For these reasons, we rejected this model in favour of the model presented in the main text.

## Text S6

### Relative OXPHOS contribution to power supply

It is interesting to observe the relative contributions of oxidative phosphorylation and glycolysis to power supply. Since Eq.(9) states that power supply = demand, where demand corresponds to cell volume, the ratio $f_o = k_o P^+/V$ determines the relative contribution of OXPHOS to power supply, see Figure S15.

For $h < h^*$, we see that OXPHOS has decreasing contributions to power supply. At $h^*$, OXPHOS contributions stabilize with $0.28 < f_o(h = 0.52 \approx h^*_{\mathrm{MAP}}) < 0.44$ (25-75% CI). The heteroplasmy at which OXPHOS contributions are stabilized corresponds to the hypothesized demand/supply toggle, where the cell attempts to increase power supply as opposed to reducing power demand.

The value of $f_o$ where OXPHOS contributions become stabilized ($f_o(h^*)$) may have wider significance. Mitochondrial metabolism, and especially mitochondrial membrane potential, is connected to a variety of biosynthetic pathways [1] and crucial for maintaining cellular proliferation [14]. $f_o(h^*)$ may represent a minimum ETC flux, relative to power demand, for mitochondria to support their mitochondrial membrane potential without the aid of glycolytic ATP. Below $f_o(h^*)$, we might predict that cells run ATP synthase in reverse, hydrolysing glycolytic ATP to maintain membrane potential.

# References


1. Hastings, W.K. (1970) Monte Carlo sampling methods using Markov chains and their applications *Biometrika* **57**, 97–109

2. Picard, M., Zhang, J., Hancock, S., Derbeneva, O., Golhar, R., Golik, P., O'Hearn, S., Levy, S., Potluri, P., Lvova, M. et al. (2014) Progressive increase in mtDNA 3243A > G heteroplasmy causes abrupt transcriptional reprogramming *Proc. Natl Acad. Sci. USA* **111**, E4033–E4042

3. Haario, H., Saksman, E., and Tamminen, J. (2001) An adaptive Metropolis algorithm *Bernoulli* **7**, 223–242

4. Elowitz, M.B., Levine, A.J., Siggia, E.D., and Swain, P.S. (2002) Stochastic gene expression in a single cell *Science* **297**, 1183–1186

5. Chujo, T., Ohira, T., Sakaguchi, Y., Goshima, N., Nomura, N., Nagao, A., and Suzuki, T. (2012) LRPPRC/SLIRP suppresses PNPase-mediated mRNA decay and promotes polyadenylation in human mitochondria *Nucleic Acids Res.* **40**, 8033–8047

6. Ojala, D., Montoya, J., and Attardi, G. (1981) tRNA punctuation model of RNA processing in human mitochondria *Nature* **290**, 470–474

7. Buttgereit, F., Brand, M., and Müller, M. (1992) ConA induced changes in energy metabolism of rat thymocytes *Biosci. Rep.* **12**, 109–114

8. Brand, M.D., Couture, P., Else, P.L., Withers, K.W., and Hulbert, A. (1991) Evolution of energy metabolism. Proton permeability of the inner membrane of liver mitochondria is greater in a mammal than in a reptile *Biochem. J.* **275**, 81–86

9. Rolfe, D. and Brown, G.C. (1997) Cellular energy utilization and molecular origin of standard metabolic rate in mammals *Physiol. Rev.* **77**, 731–758

10. Alberts, B., Johnson, A., Lewis, J., Roberts, K., Raff, M., and Walter, P. (2002) *Molecular Biology of the Cell* Garland Science

11. Wasserman, L. (2013) *All of Statistics: A Concise Course in Statistical Inference* Springer Texts in Statistics Springer New York

12. Tzur, A., Kafri, R., LeBleu, V.S., Lahav, G., and Kirschner, M.W. (2009) Cell growth and size homeostasis in proliferating animal cells *Science* **325**, 167–171

13. Neumann, F.R. and Nurse, P. (2007) Nuclear size control in fission yeast *J. Cell Biol.* **179**, 593–600

14. Schuler, M., Muehlbauer, P., Guzzie, P., and Eastmond, D. (1999) Noscapine hydrochloride disrupts the mitotic spindle in mammalian cells and induces aneuploidy as well as polyploidy in cultured human lymphocytes *Mutagenesis* **14**, 51–56

15. Bereiter-Hahn, J. and Vöth, M. (1998) Distribution and dynamics of mitochondrial nucleoids in animal cells in culture in *Experimental Biology Online* Springer 58–77

16. Alán, L., Zelenka, J., Ježek, J., Dlasková, A., and Ježek, P. (2010) Fluorescent in situ hybridization of mitochondrial DNA and RNA *Acta Biochim. Pol.* **57**, 403

17. Ozawa, T., Natori, Y., Sato, M., and Umezawa, Y. (2007) Imaging dynamics of endogenous mitochondrial RNA in single living cells *Nature Methods* **4**, 413–419

18. Chatre, L. and Ricchetti, M. (2013) Large heterogeneity of mitochondrial DNA transcription and initiation of replication exposed by single-cell imaging *J. Cell Sci.* **126**, 914–926

19. Busch, K.B., Kowald, A., and Spelbrink, J.N. (2014) Quality matters: how does mitochondrial network dynamics and quality control impact on mtDNA integrity? *Phil. Trans. R. Soc. B* **369**, 20130442

20. Wilkens, V., Kohl, W., and Busch, K. (2013) Restricted diffusion of OXPHOS complexes in dynamic mitochondria delays their exchange between cristae and engenders a transitory mosaic distribution *J Cell Sci* **126**, 103–116

21. Sakaue-Sawano, A., Kurokawa, H., Morimura, T., Hanyu, A., Hama, H., Osawa, H., Kashiwagi, S., Fukami, K., Miyata, T., Miyoshi, H. et al. (2008) Visualizing spatiotemporal dynamics of multicellular cell-cycle progression *Cell* **132**, 487–498

22. Ahn, C.S. and Metallo, C.M. (2015) Mitochondria as biosynthetic factories for cancer proliferation *Cancer Metab* **3**, 1 doi:10.1186/s40170-015-0128-2

23. Martínez-Reyes, I., Diebold, L.P., Kong, H., Schieber, M., Huang, H., Hensley, C.T., Mehta, M.M., Wang, T., Santos, J.H., Woychik, R. et al. (2015) TCA Cycle and Mitochondrial Membrane Potential Are Necessary for Diverse Biological Functions *Mol. Cell* **61**, 199–209

24. Sasarman, F., Antonicka, H., and Shoubridge, E.A. (2008) The A3243G tRNALeu (UUR) MELAS mutation causes amino acid misincorporation and a combined respiratory chain assembly defect partially suppressed by overexpression of EFTu and EFG2 *Hum. Mol. Genet.* **17**, 3697–3707

25. Whitfield, M.L., Sherlock, G., Saldanha, A.J., Murray, J.I., Ball, C.A., Alexander, K.E., Matese, J.C., Perou, C.M., Hurt, M.M., Brown, P.O. et al. (2002) Identification of genes periodically expressed in the human cell cycle and their expression in tumors *Mol. Biol. Cell* **13**, 1977–2000

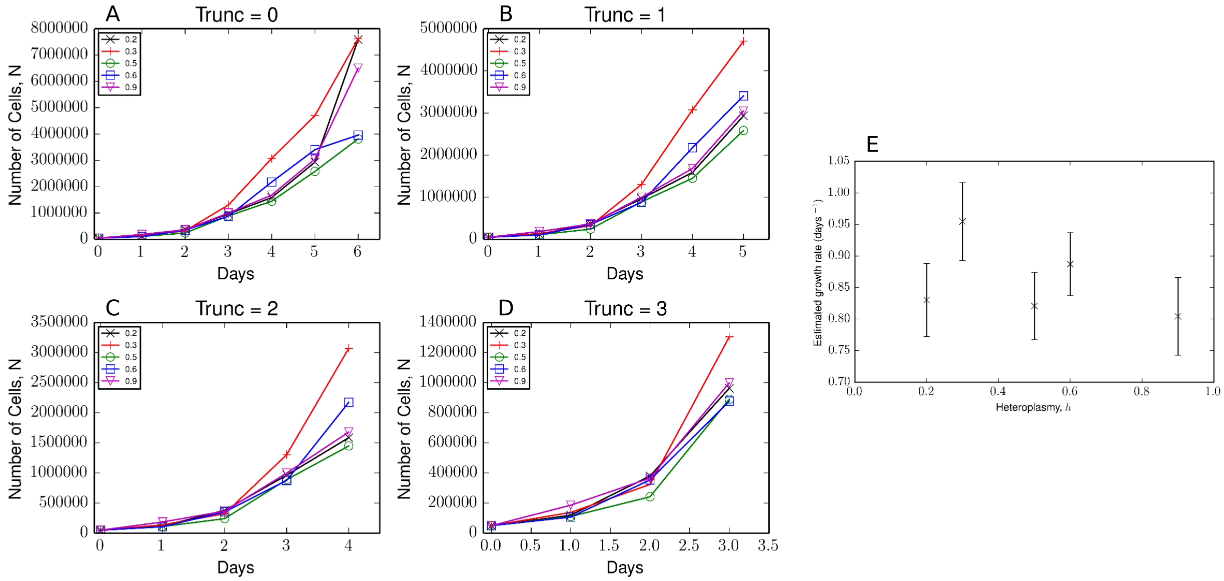


**Figure S1. Cell proliferation data from [13].** A-D. Number of cells ($N$) versus number of days of incubation, for different heteroplasmies, where a number of data points have been truncated (Trunc) from the right. Growth appears to be non-exponential by day 6, and is therefore removed subsequently. E. Slope of linear regression with associated standard error, to derive the growth rate in dimensions of days$^{-1}$, as used in Fig. 1E. Raw data provided by Martin Picard.

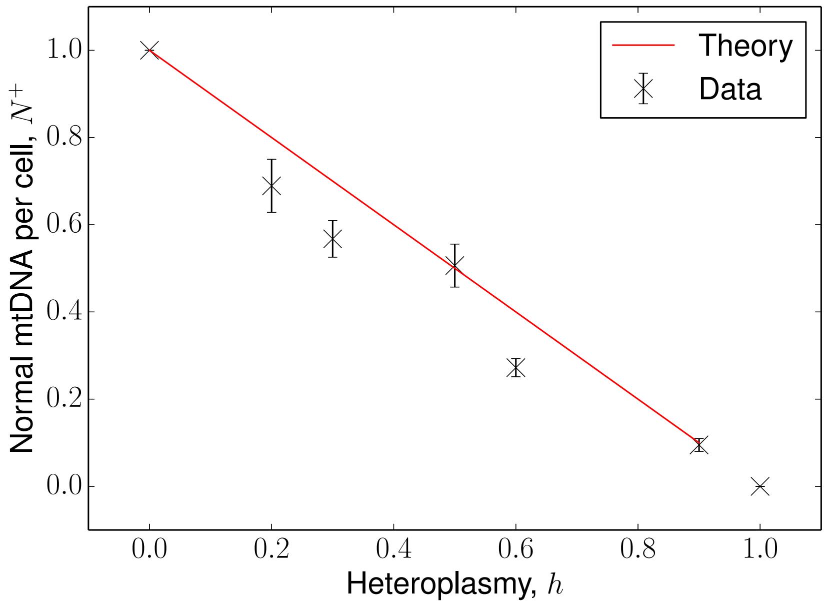


**Figure S2. Model fit for wild-type mtDNA copy number** Data for mtDNA copy number from Picard *et al.* [13] was multiplied by (1-h), as was the SEM. Displaying the model $N^+ = N(1-h)$, for $N = \text{const} = 1$.

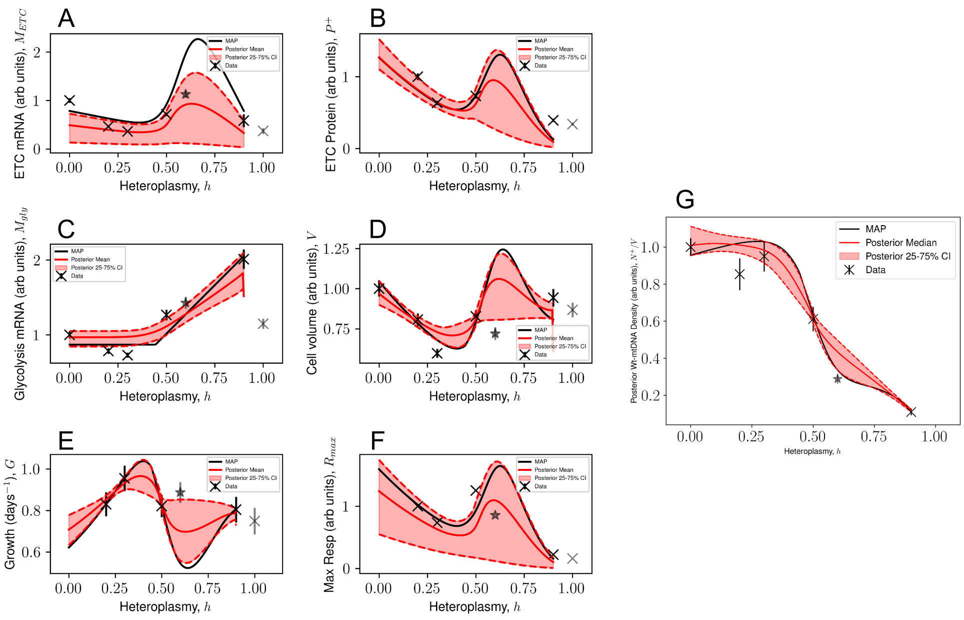


**Figure S3. Model fits are largely robust to exclusion of data at 60% heteroplasmy**. All data, if present, for $h = 0.6$ (grey stars) were excluded and the model was re-fitted as per Text S2. (A-F) We observe the same qualitative behaviour as Fig. 3. $M_{\mathrm{ETC}}$ fits are expected to display a peak around $h = 0.6$ (see posterior mean), as is $P^+$. $M_{\mathrm{gly}}$ is essentially unaffected, whereas $V$, $G$ and $R_{\mathrm{max}}$ also show exaggerated behaviour relative to Fig. 3 at $h = 0.6$. (G) The mean posterior density of $N^+/V$ is approximately constant between $0 \leq h \leq 0.3$, as seen in Fig. 4, suggesting that the hypothesis of wild-type density homeostasis is robust under the exclusion of this data point, given our model structure and priors.

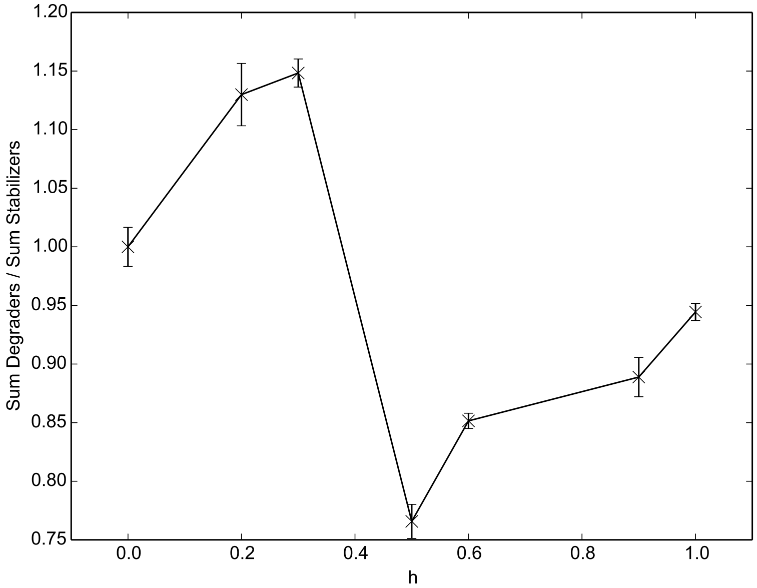


**Figure S4. Corroborating evidence for ETC mRNA degradation from Picard *et al*.** Ratio of mitochondrial mRNA degraders (PDE12, PNPT1, SUPV3L1) to stabilizers (MTPAP, LRPPRC, SLIRP), see Ref. [28] and references therein. We observe qualitative similarity in $\delta_m$ (see Fig. 5) and the ratio of normalised genes, both showing strong downregulation between $h = 0.3$ and $h = 0.5$. The numerator and denominator were normalised according to Eq.(1). Errors result from error propagation of a ratio, where the error for the numerator and denominator are derived using Eq.(2).

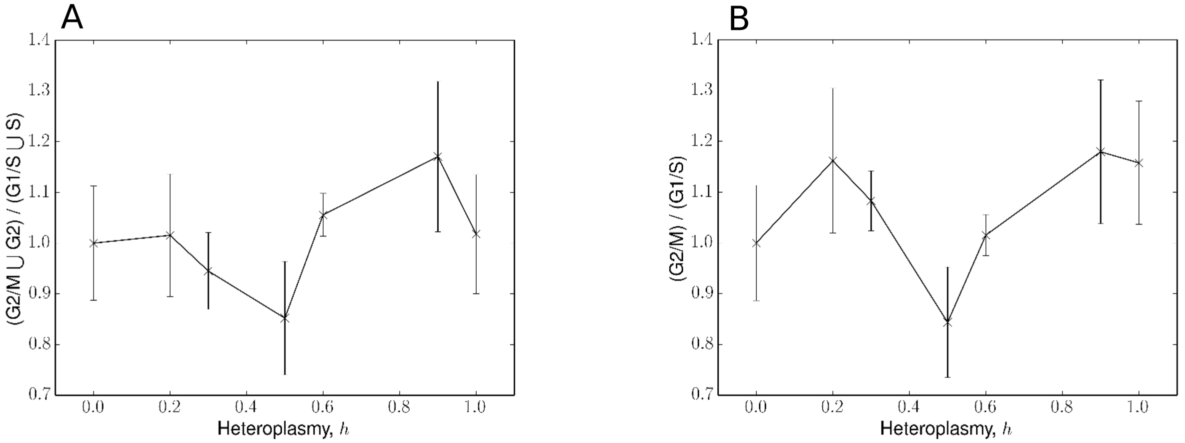


**Figure S5. Variation of cell cycle markers with heteroplasmy** A list of cell cycle markers, taken from [45] were normalised according to Eq.(1), yielding gene lists for G1/S, S, G2 and G2/M phases. A. The ratio of G2/M to G1/S genes yielded no obvious trend with heteroplasmy. B. G2/M and G2 gene lists were combined (G2/M $\bigcup$ G2), as were G1/S and S (G1/S $\bigcup$ S). This again yielded no obvious trend with heteroplasmy. Errors result from error propagation of a ratio, where the error for the numerator and denominator are derived using Eq.(2).

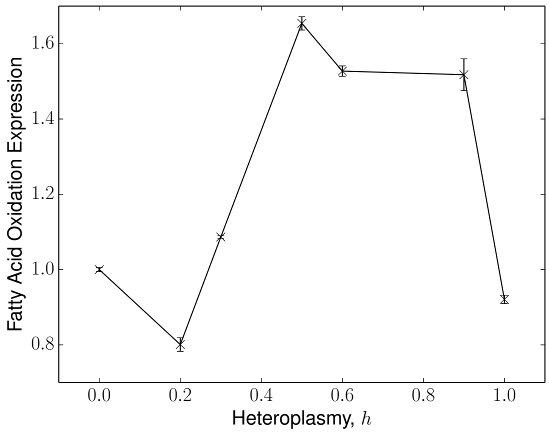


**Figure S6. Expression of fatty acid oxidation enzymes with heteroplasmy**. Showing variation of the genes (ACADVL, ECHS1, HADH and ACAA2) with heteroplasmy, normalised according to Eq.(1). It can be seen that these metabolites are downregulated between $h = 0.9 \rightarrow 1$, so fatty acid oxidation does not appear to be supporting the maintained cell volume and growth rates over this range of heteroplasmy. Errors are calculated using Eq.(2).

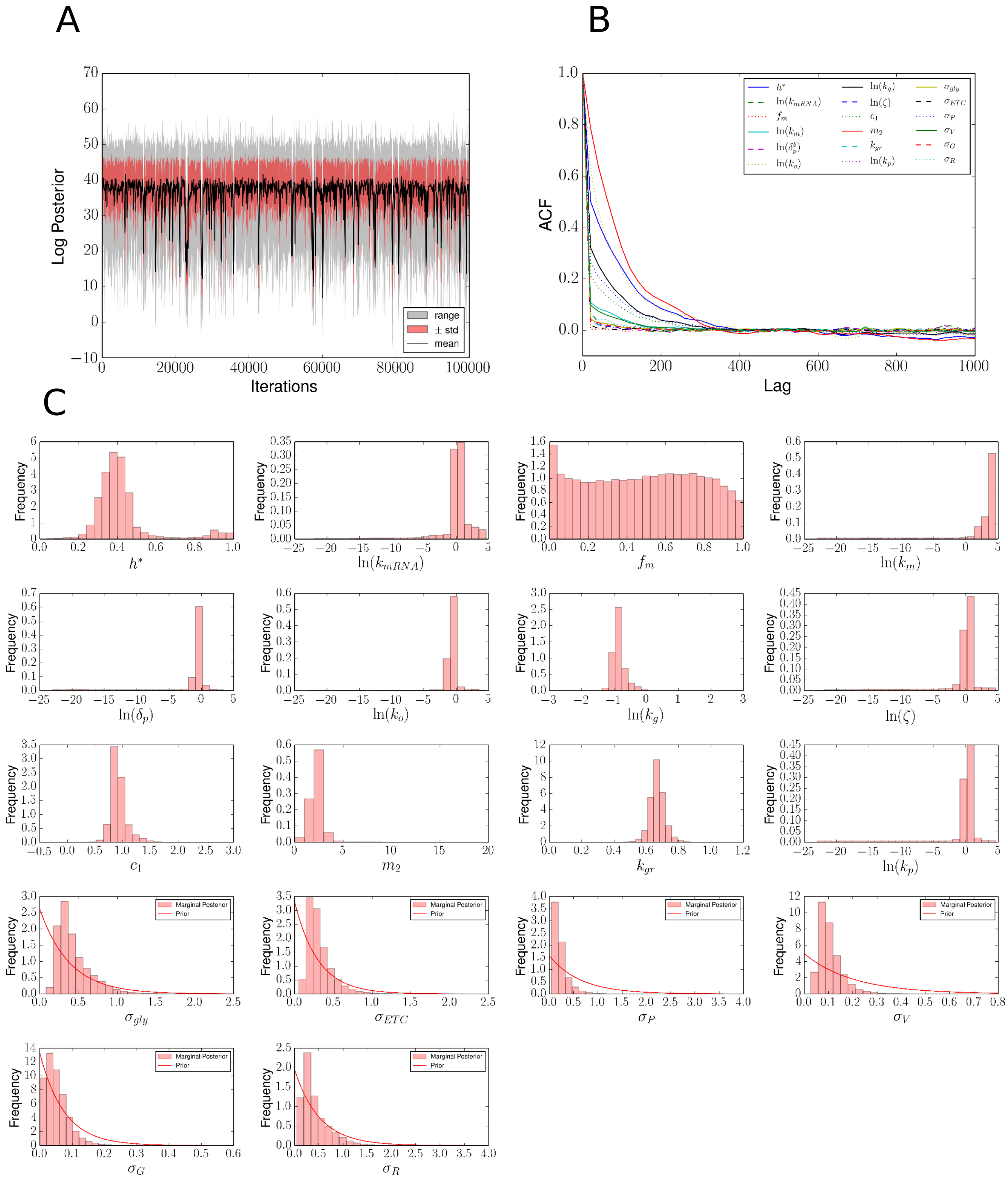


**Figure S7. Metropolis-Hastings posterior samples for main model** $10^{10}$ iterations of Metropolis-Hastings were performed, which were thinned to $10^5$ samples. The hyperparameter for model uncertainty, $\Omega$, was chosen as $\Omega = 2$ (see Text S2). A. Trajectory of the unnormalised log posterior after thinning. Samples are split into bins of 100 iterations; displaying mean, standard deviation and range of each bin. B. Autocorrelation function for each parameter, on thinned samples. C. Approximate marginal posterior distributions for each parameter in the model. Exponential priors for model uncertainties are plotted, all other priors are uniform (see Text S2).

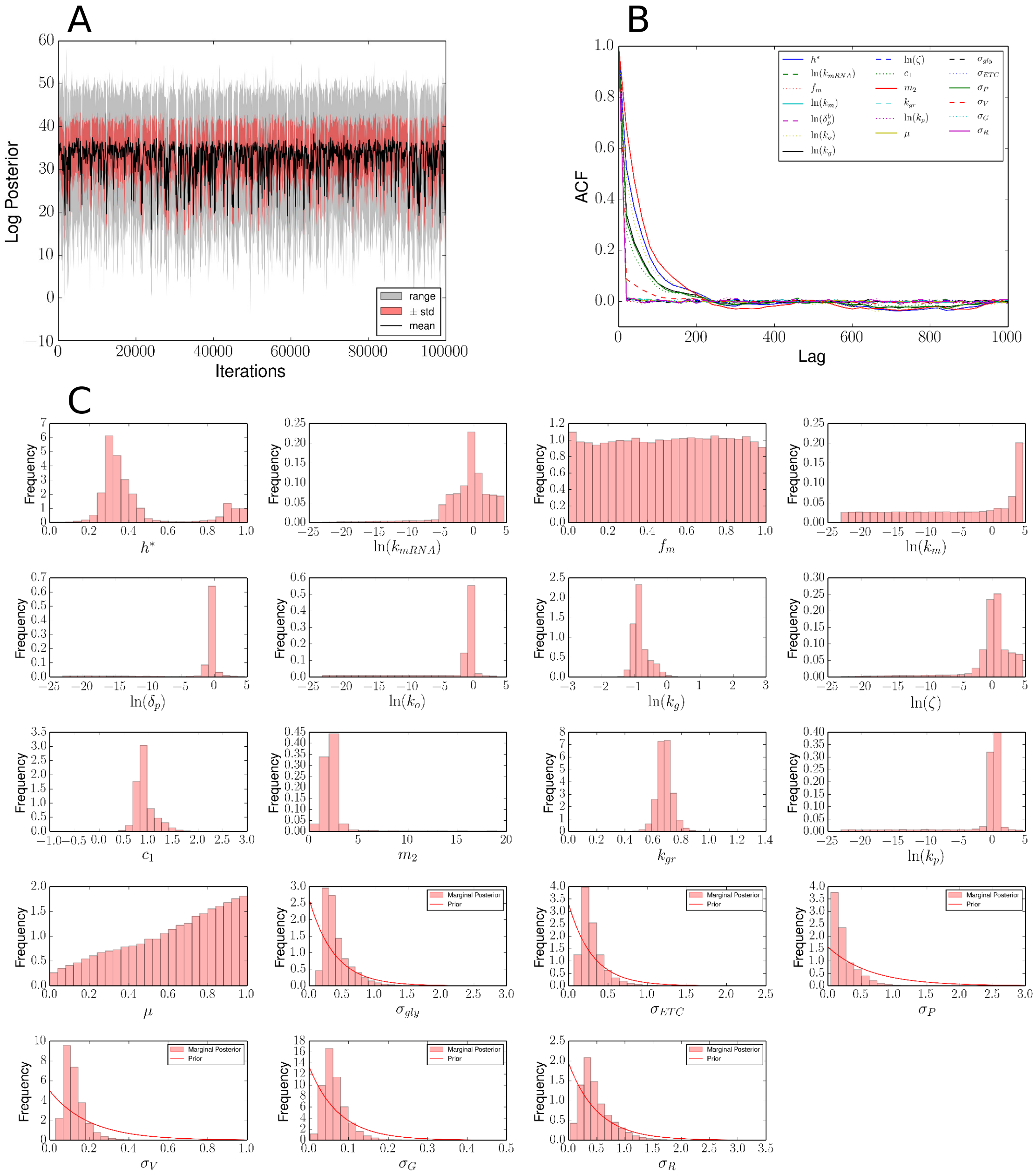


**Figure S8. Metropolis-Hastings posterior samples for mutant transcription model** $10^{10}$ iterations of Metropolis-Hastings were performed, which were thinned to $10^5$ samples. The hyperparameter for model uncertainty, $\Omega$, was chosen as $\Omega = 2$ (see Text S2). The prior for the additional parameter $\mu$ was chosen to be a uniform distribution between 0 and 1. A. Trajectory of the unnormalised log posterior after thinning. Samples are split into bins of 100 iterations; displaying mean, standard deviation and range of each bin. B. Autocorrelation function for each parameter, on thinned samples. C. Approximate marginal posterior distributions for each parameter in the model. Exponential priors for model uncertainties are plotted, all other priors are uniform (see Text S2). Note the marginal distribution of $\mu$ peaks near 1.

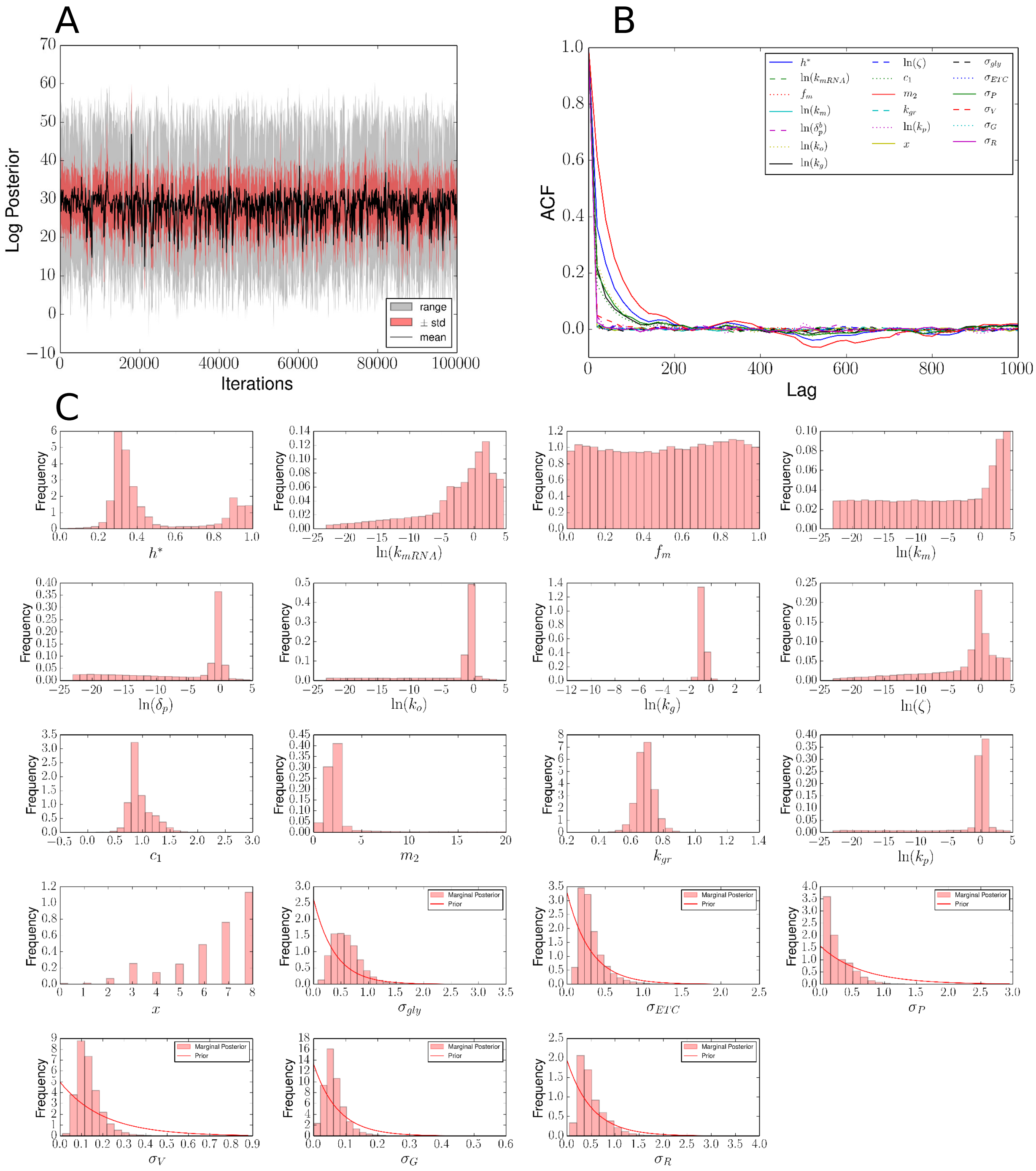


**Figure S9. Metropolis-Hastings posterior samples for tRNA misincorporation model** $10^{10}$ iterations of Metropolis-Hastings were performed, which were thinned to $10^5$ samples. The hyperparameter for model uncertainty, $\Omega$, was chosen as $\Omega = 2$ (see Text S2). The prior for the additional parameter $x$ was chosen to be a uniform discrete distribution between 0 and 8. A. Trajectory of the unnormalised log posterior after thinning. B. Autocorrelation function for each parameter, on thinned samples. C. Approximate marginal posterior distributions for each parameter in the model. Exponential priors for model uncertainties are plotted, all other priors are uniform (see Text S2). Note the marginal distribution of $x$ peaks at 8, which we expect to be incorrect.

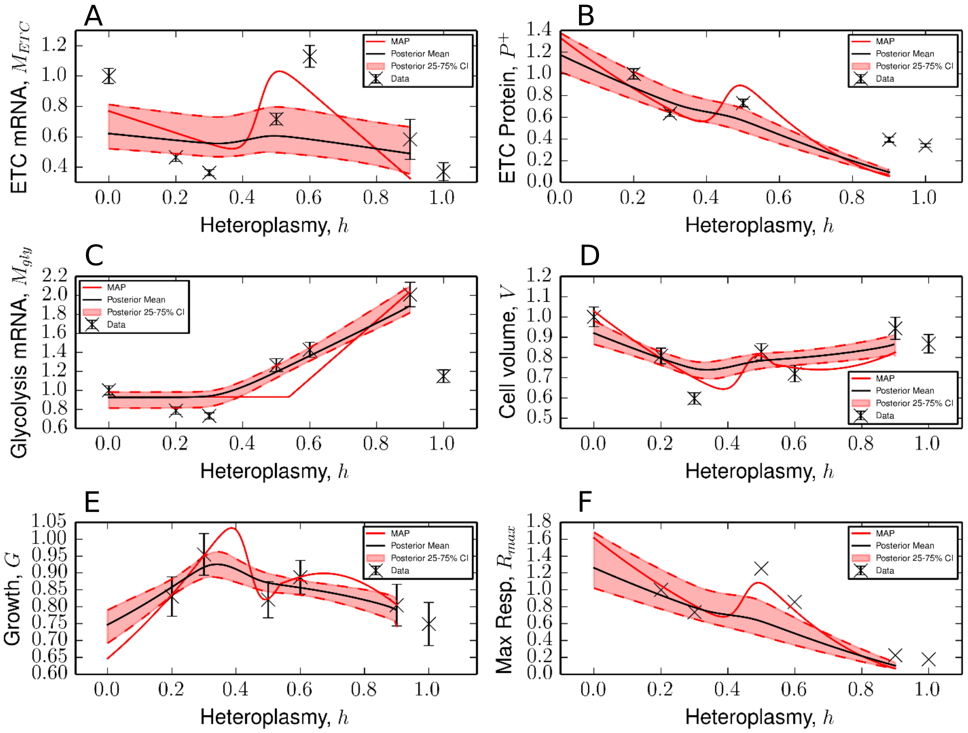


**Figure S10. Alternative mutant transcription model**. A-F. Model fit when Eq.(5) is replaced with Eq.(S34). The 25-75% CI is flatter when compared with Fig. 3. We find this is due to the model more frequently selecting linear fits to the data, see Figure S16.

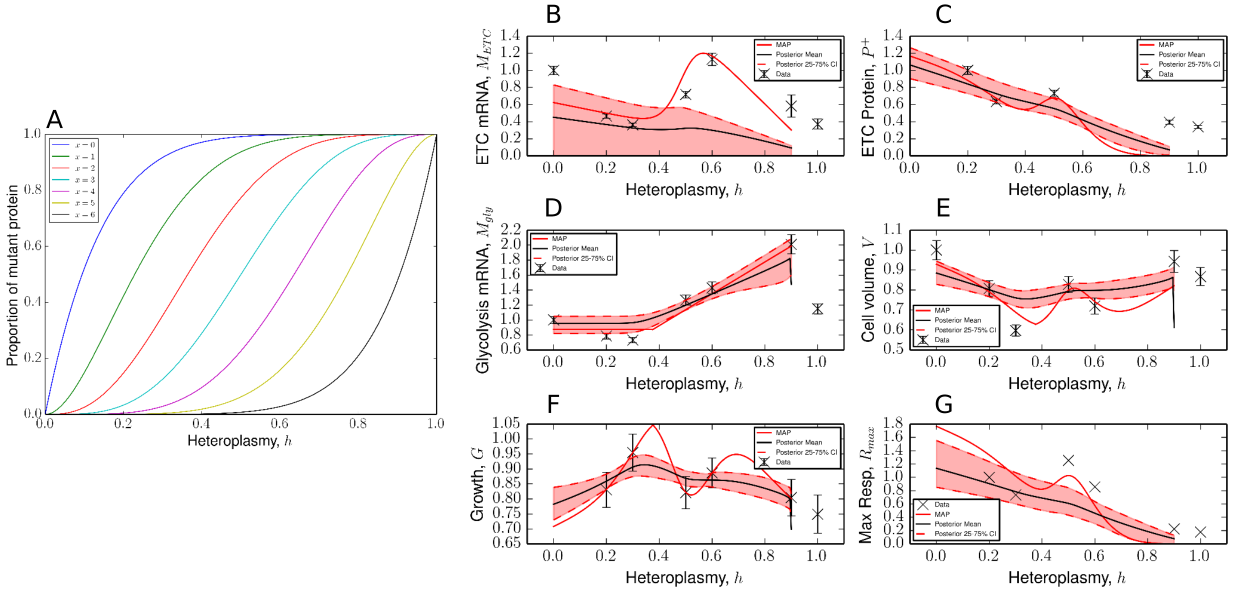


**Figure S11. Alternative tRNA misincorporation model** A. Expected proportion of mutant protein due to the MELAS mutation, given that each protein can tolerate $x$ mutated residues. The chain length used is $N = 8$, which is the mean number of susceptible residues across all mitochondrially-encoded peptides, excluding ATP8 and ATP6 [9]. B-G. Model fit when Eq.(7) is replaced with Eq.(S37). $M_{\mathrm{ETC}}$ is qualitatively fitted more poorly than Fig. 3 (see also Figure S16), although the maximum a posteriori estimate is a closer fit, when compared with Fig. 3.

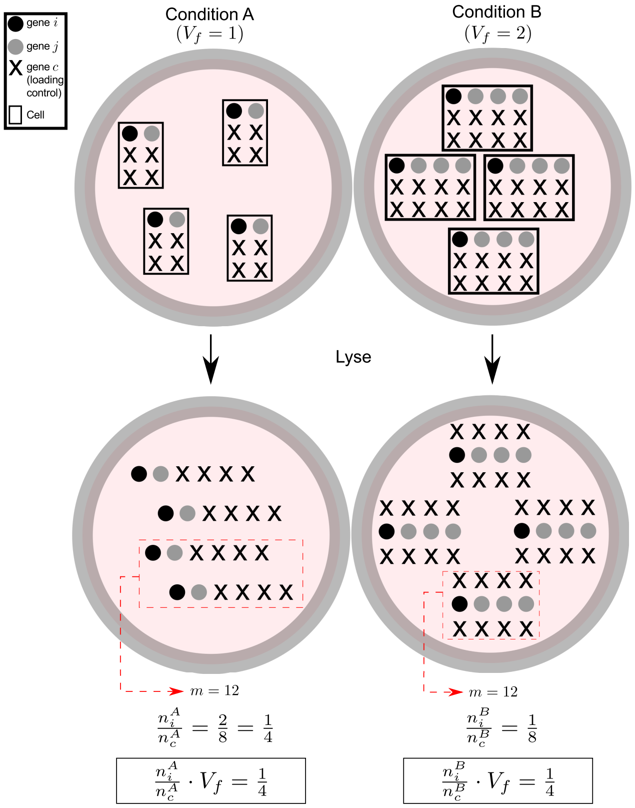


**Figure S12. Transformation of Western blot (or RNA-seq) data to per-cell dimensions.** Consider a Western blot experiment, where we are interested in the fold-change expression of gene $i$ per cell ($n_i$), and cell volume has a fold change $V_f = 2$ between conditions A and B. In this example, $n_i^A = n_i^B$. Taking an unbiased sample of size $m = 12$ from each condition, and dividing by the loading control, yields a quantity $1/V_f$ too small. It is necessary to multiply the ratio by $V_f$, to get an accurate measurement of gene $i$, in the context of strongly varying cell volume, as is the case in Picard *et al.* [13]. A similar argument holds for RNA-seq data, which also uses a fixed mass of RNA as the starting sample.

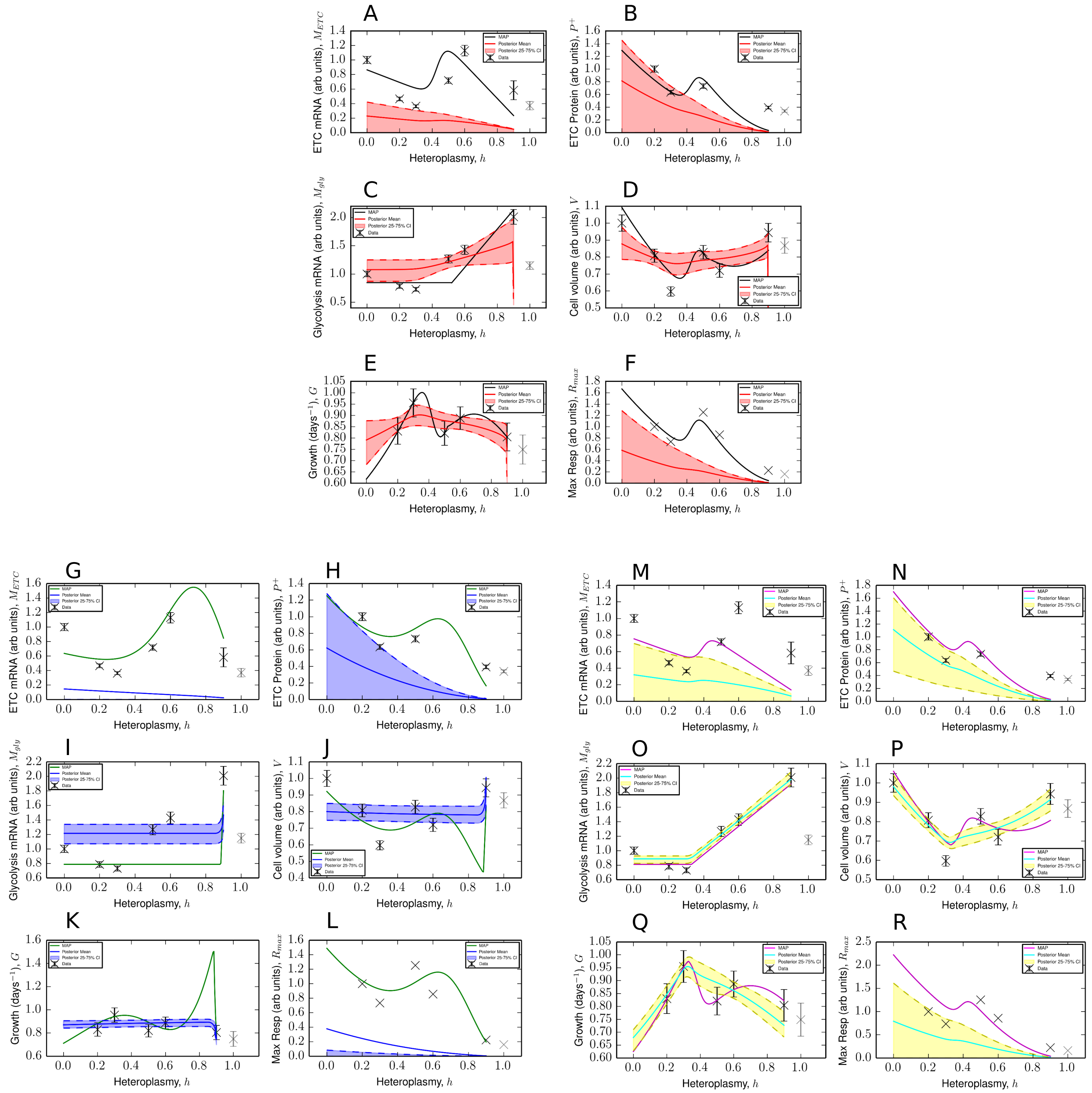


**Figure S13. Hyperparameter choice for error model** A-F: Model support for when $\Omega = 0$, corresponding to an improper uniform prior on the variance of each feature. G-L: Sub-samples of posterior where $0.85 \leq h^* \leq 0.9$. M-R: Sub-samples of posterior where $0.3 \leq h^* \leq 0.35$. We see a physiologically implausible fit for $0.85 \leq h^* \leq 0.9$, whereas when $0.3 \leq h^* \leq 0.35$ model fits were better able to describe the data (for instance, by comparing G to M).

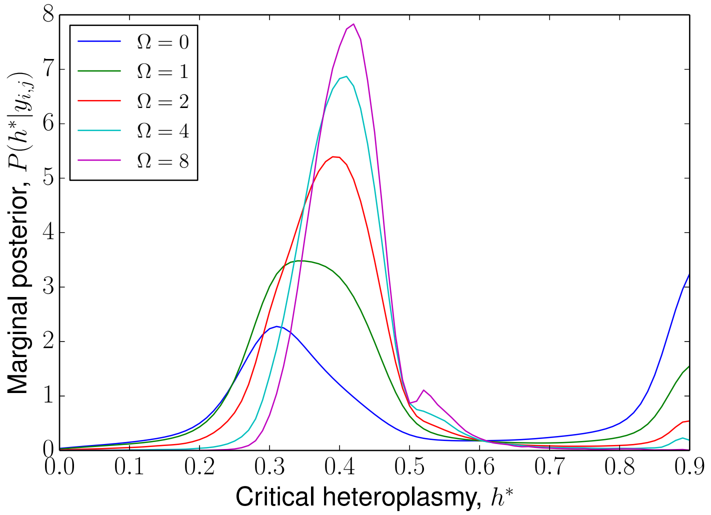


**Figure S14. Choice of uncertainty prior affects distribution of critical heteroplasmy and model fit** Larger values of $\Omega$ suppresses large values of model uncertainty ($\sigma_i$, see Eq.(S10)), and consequently forces the model fit to more closely match the data. This corresponds to the $h^*$ mode approximately between 0.3-0.4 see Figure S13.

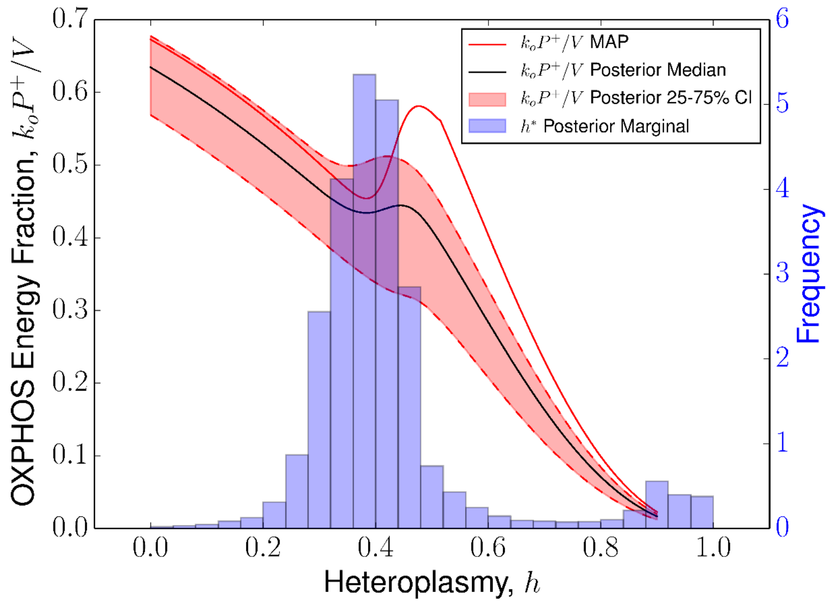


**Figure S15. Relative contribution of OXPHOS to total power supply across heteroplasmy** Posterior statistics for the ratio of OXPHOS power supply ($k_oP^+$) to total power supply $k_oP^+ + k_gM_{\text{gly}} = V$. The contribution of ETC power production reduces until the critical heteroplasmy $h^*$, where a compensatory response stabilizes OXPHOS contributions. As $\delta_m \to 0$ at $h \approx 0.5$ (see Fig. 5), OXPHOS power contributions continue to diminish.

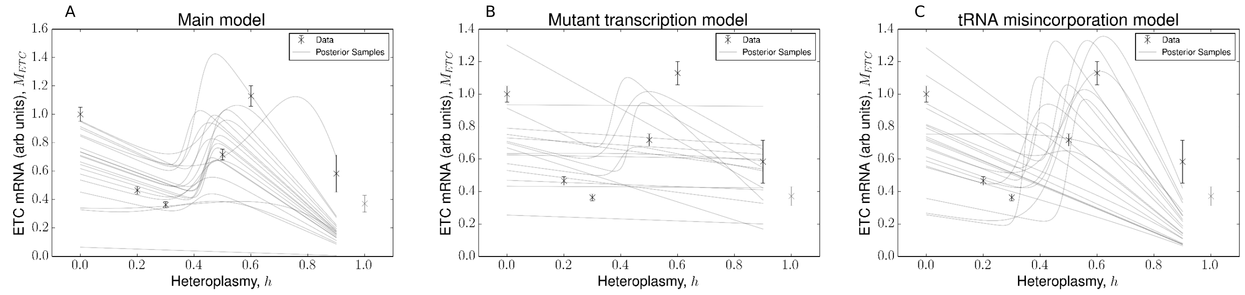


**Figure S16. Comparison of ETC mRNA levels for alternative models**. Sample of 20 randomly sampled trajectories from the posterior distribution of $M_{ETC}$ for A. Main model (see Interactions between Bioenergetic Variables can be Cast as a Bottom-up Quantitative Model); B. Mutant transcription model (see Text S5); C. tRNA misincorporation model (see Text S5). In models (B) and (C), we find that linear fits are more frequently selected when compared to our main model (A), despite being more complex in terms of number of parameters.

**Table S1.** Table of observables, and their corresponding parameters

| Observables | Parameter | Description |
|---|---|---|
| $M_{ETC}$ and $M_{gly}$ | $h^*$ | Critical heteroplasmy |
| $M_{ETC}$ | $k_{mRNA}$ | Maximum ETC mRNA degradation rate |
| | $f_m$ | Relative ETC mRNA degradation rate at $h^*$ |
| | $k_m$ | ETC mRNA degradation steepness |
| | $\zeta$ | $\beta/k_{mRNA}$ where $\beta$ = ETC mRNA transcription rate |
| $P^+$ | $\delta_p$ | ETC protein passive degradation rate |
| $M_{gly}$ | $c_1$ | Glycolysis mRNA pool size for $h \leq h^*$ |
| | $m_2$ | Glycolysis rate of increase with $h$, for $h > h^*$ |
| $V$ | $k_o$ | ETC protein constant of proportionality |
| | $k_g$ | Glycolysis mRNA constant of proportionality |
| $G$ | $k_{gr}$ | Constant of proportionality |
| $R_{max}$ | $k_p$ | Constant of proportionality |

**Table S2. Summary of experimental proposals, corresponding to the claims of the model**.

| Claim | Experiment |
|---|---|
| MtDNA copy number affects volume | Perturb mtDNA copy number (ddC or PGC1-$\alpha$), measure volume |
| Wild-type mtDNA density affects $h^*$ (bioenergetic) | Increase nDNA content, perform RNA-seq |
| Wild-type mtDNA density affects $h^*$ (mechanical) | Reduce cell volume, perform RNA-seq |
| Mutant mtDNAs have transcription defect | Measure normal/mutant tRNA abundance with RT-qPCR |
| Mitochondrial tRNAs have low diffusivity | Fluorescent labelling of mtDNA and tRNA or mRNA encoding tRNA |
| Cell cycle variation with heteroplasmy | Fucci markers in heteroplasmic cells |
| Mean cell volume affects growth rate | Synchronise, sort by volume, and measure growth rate |
| Maximum respiratory capacity $\propto$ ETC protein | Measure $R_{\max}$ at $h = 0$ and $P^+$ at $h = 0.0, 0.6$ |
